# Cell-intrinsic metabolic phenotypes identified in patients with glioblastoma, using mass spectrometry imaging of ^13^C-labelled glucose metabolism

**DOI:** 10.1038/s42255-025-01293-y

**Published:** 2025-05-19

**Authors:** Anastasia Tsyben, Andreas Dannhorn, Gregory Hamm, Manthos Pitoulias, Dominique-Laurent Couturier, Ashley Sawle, Mayen Briggs, Alan J. Wright, Cara Brodie, Lee Mendil, Jodi L. Miller, Eleanor C. Williams, Lovisa Franzén, Grand De Jong, Tannia Gracia, Fani Memi, Omer Ali Bayraktar, Ram Adapa, Jyotsna Rao, Ariadna González-Fernández, CRUK Rosetta Grand Challenge Consortium, Josephine Bunch, Zoltan Takats, Simon T. Barry, Richard J. A. Goodwin, Richard Mair, Kevin M. Brindle

**Affiliations:** 1 Cancer Research UK Cambridge Institute, University of Cambridge, Cambridge, UK; 2 Integrated BioAnalysis, Clinical Pharmacology & Safety Sciences R&D, AstraZeneca, Cambridge, UK; 3 AstraZeneca, Cambridge Biomedical Campus, Cambridge, UK; 4 Cambridge Stem Cell Institute, University of Cambridge, Cambridge, UK; 5 Safety Sciences, Clinical Pharmacology & Safety Sciences R&D, AstraZeneca, Gothenburg, Sweden; 6 Department of Gene Technology, KTH Royal Institute of Technology, Science for Life Laboratory, Stockholm, Sweden; 7 Wellcome Sanger Institute, Wellcome Genome Campus, Hinxton, Cambridge, UK; 8 National Physical Laboratory, Teddington, UK; 9 Department of Metabolism, Digestion and Reproduction, Imperial College, London, UK; 10 Department of Clinical Neurosciences, Cambridge Biomedical Campus, Cambridge, UK; 11 Department of Biochemistry, University of Cambridge, Cambridge, UK

**Keywords:** Cancer metabolism, Mass spectrometry, Metabolism

## Abstract

Transcriptomic studies have attempted to classify glioblastoma (GB) into subtypes that predict survival and have different therapeutic vulnerabilities^1–3^. Here we identified three metabolic subtypes: glycolytic, oxidative and a mix of glycolytic and oxidative, using mass spectrometry imaging of rapidly excised tumour sections from two patients with GB who were infused with [U-^13^C]glucose and from spatial transcriptomic analysis of contiguous sections. The phenotypes are not correlated with microenvironmental features, including proliferation rate, immune cell infiltration and vascularization, are retained when patient-derived cells are grown in vitro or as orthotopically implanted xenografts and are robust to changes in oxygen concentration, demonstrating their cell-intrinsic nature. The spatial extent of the regions occupied by cells displaying these distinct metabolic phenotypes is large enough to be detected using clinically applicable metabolic imaging techniques. A limitation of the study is that it is based on only two patient tumours, albeit on multiple sections, and therefore represents a proof-of-concept study.

## Abstract (web summary)

Tsyben et al. study the spatial distribution of labelled metabolites in glioblastoma samples from patients infused with ^13^C-glucose, using mass spectrometry imaging.

## Main

GB is the most common primary adult brain cancer^4^. Transcriptomic analyses have attempted to classify GB into subtypes that could predict treatment response^1–3^, and a recent study that used a pathway-based classification defined metabolism-associated subtypes with distinct therapeutic vulnerabilities. These included a mitochondrial subtype, which is associated with a more favourable clinical outcome and is sensitive to inhibitors of oxidative phosphorylation, and a glycolytic, plurimetabolic subtype that is resistant to multiple treatment types^5^.

An important question is the extent to which the metabolism displayed by tumour cells in vivo is cell-intrinsic and how much is defined by the tumour microenvironment (TME)^6^. We addressed this question by using mass spectrometry imaging (MSI) of rapidly excised tumour sections from patients with GB who were infused with [U-^13^C]glucose immediately before surgery to image tumour cell metabolic activity in vivo and from a spatial transcriptomic analysis of adjacent sections.

## Results

**Fig. 1:**
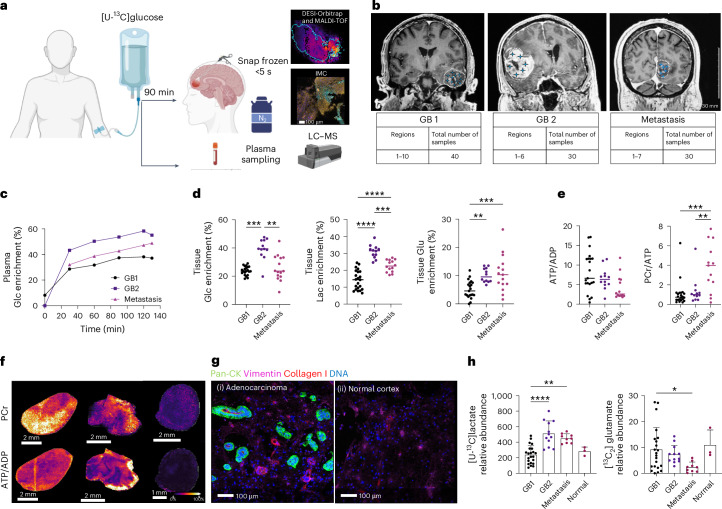
Intra-operative freezing rapidly arrests tissue metabolism and allows visualisation of metabolic activity. **a**, Patients were infused intra-operatively with [U-^13^C]glucose and underwent multi-regional tumour sampling followed by rapid freezing in liquid nitrogen (<5 s). Tumour sections (10 μm) were analysed using DESI-MSI and MALDI-MSI, and contiguous sections were analysed by IMC. Blood samples were collected before, during and after infusion for plasma liquid chromatography–MS (LC–MS) analysis. Created in BioRender.com. **b**, Coronal MR images from the three patients who were infused, showing the locations of the sampled regions (blue stars). The number of regions sampled is shown for each patient; each region had three to six pieces of snap-frozen tissue. **c**, Plasma glucose (Glc) fractional labelling in the three infused patients. Tumour sampling commenced at 90 min. **d**, Glucose and metabolite fractional labelling in the tumour tissue: Glc ([U-^13^C]glucose/([U-^13^C]glucose + [U-^12^C]glucose) (GB1 vs GB2, *P* = 0.0001; GB2 vs Metastasis, *P* = 0.004); lactate (Lac) ([U-^13^C]lactate/([U-^13^C]lactate + [U-^12^C]lactate) (GB1 vs GB2, *P* < 0.0001; GB2 vs Metastasis, *P* = 0.0002; GB1 vs Metastisis, *P* < 0.0001); and glutamate (Glu) ([^13^C_2_]glutamate/([^13^C_2_]glutamate + [U-^12^C]glutamate) (GB1 vs GB2, *P* = 0.0069; GB1 vs Metastasis, *P* = 0.0008). **e**, ATP/ADP and PCr signal intensity ratios in each tumour section (PCr/ATP GB1 vs Metastasis, *P* = 0.0001; GB2 vs Metastasis, *P* = 0.0033). **f**, Representative tumour sections from each patient showing PCr signal intensities and ATP/ADP signal intensity ratios. **g**, Representative sections from the metastasis and from normal-appearing brain (data reproduced in three additional sections of normal cortex and nine of the metastasis); (i) Pan-cytokeratin (CK)-positive (green) adenocarcinoma cells formed tubules around a central Collagen I-positive vessel (red). The tubules are surrounded by vimentin-positive (pink) cortex. (ii) Normal-appearing cortical tissue was obtained from the margin of the metastasis and showed vimentin-positive cells and an absence of adenocarcinoma cells. **h**, Relative abundance of [U-^13^C]lactate (GB1 vs GB2, *P* < 0.0001; GB1 vs Metastasis, *P* = 0.0016) and [^13^C_2_]glutamate (GB1 vs Metastasis, *P* = 0.0394) in tumour sections from GB1 (*n* = 22), GB2 (*n* = 12), the adenocarcinoma metastasis (*n* = 9) and normal-appearing cortex (*n* = 3). Data are means; error bars, s.d.; every dot is a tissue section. Asterisks refer to *P* values obtained from a one-way ANOVA followed by Tukey’s multiple comparisons test (*P < 0.05, **P < 0.005, ***P < 0.0005, ****P < 0.00005). Source data

**Extended Data Fig. 1:**
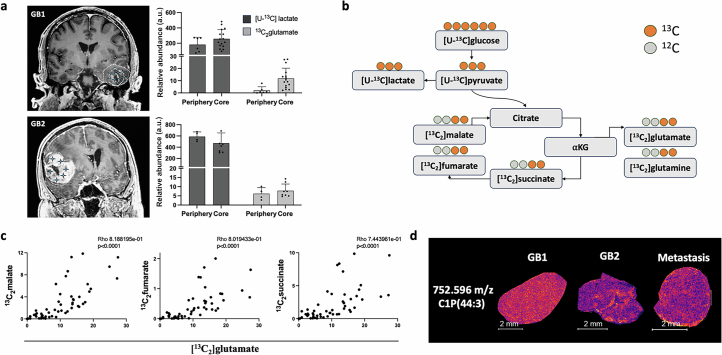
Analysis of lactate and glutamate labelling in GB1 and GB2. **a)** Coronal MR images from the two infused GB patients showing the locations of the sampled regions (blue stars). There were no significant differences in the mean signal intensities (± SD) of [^13^C_2_]glutamate and [U-^13^C]lactate in the core and periphery of GB1 (core=9; periphery =13) and GB2 (core=8; periphery =4) (unpaired T-test). **b)** Labelling of lactate and TCA cycle intermediates from [U-^13^C]glucose. **c)** Simple linear regression of the [^13^C_2_]glutamate signal intensities against the intensities of labelled TCA cycle metabolites. Each dot represents the mean intensity of the metabolite per tumour section (^13^C_2_ malate: R^2^ = .55, F(1, 51) = 62.56, p < 0.0001; ^13^C_2_ fumarate: R^2^ = .45, F(1, 51) = 41.51, p < 0.0001; ^13^C_2_ succinate: R^2^ = .39, F(1, 51) = 32.05, p < 0.0001) **d)** MALDI-MSI images of C1P(44:3) intensity across the three different patient tumours. The first section from GB1 was located at the beginning of the slide and was imaged first while the last metastasis section was located at the end of the slide and was imaged last. Tumour section locations on the slides were randomised. Source data

We infused three patients, two with GB and a third with an adenocarcinoma metastasis, with [U-^13^C]glucose and performed MSI on rapidly excised tumour tissue that was dissected during tumour debulking surgery (Fig. 1a). We sampled 16 regions from two patients with GB and seven regions from the patient with a metastasis, the latter containing samples from normal-appearing cortex (Fig. 1b). There were no significant differences in lactate and glutamate ^13^C labelling between the tumour mass and tumour margin (Extended Data Fig. 1a).

**Extended Data Fig. 2:**
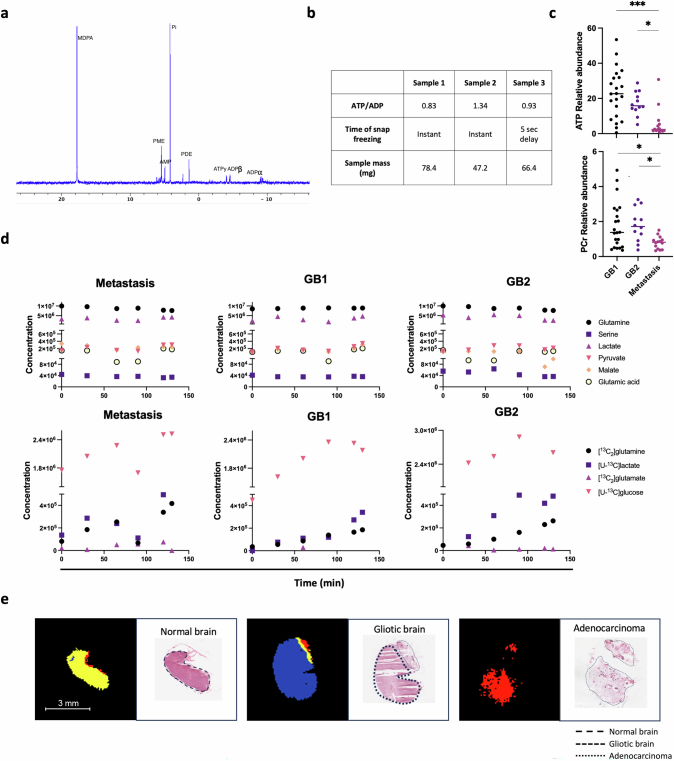
Assessment of tumour bioenergetics and analysis of plasma metabolites. **a)** ^31^P NMR spectrum of a patient’s tumour extract. The sample was collected using pituitary ronguer followed by rapid intra-operative snap freezing. **b)** ATP/ADP ratios determined from ^31^P NMR measurements on extracts from three patients tumours. The samples were frozen immediately or with 5 second delay. **c)** Relative ATP (GB1 vs met p = 0.0001; GB2 vs met p = 0.0329) and PCr (GB1 vs met p = 0.0253; GB2 vs met p = 0.047) signal intensities in each tumour section. Asterisks refer to *P* values obtained from a one way ANOVA test followed by Tukey’s multiple comparisons test. **d)** Arterial blood concentrations of unlabelled (top) and labelled (bottom) metabolites, measured using LC-MS, in the three patients during [U-^13^C]glucose infusion. **e)** K-means segmentation, based on lactate and glutamate ^13^C-labelling, of the adenocarcinoma metastasis into tumour (red), gliotic brain (blue), and normal brain (yellow). Corresponding H&E sections are shown (acquired post-DESI-MSI). Adenocarcinoma, gliotic brain and normal cortex are outlined. The H&E sections were annotated by a neuro histopathologist. Source data

Tissue sampling began 90 min after the start of infusion^7^, during which time the plasma glucose fractional enrichment reached a steady state (Fig. 1c). Fractional labelling of lactate, an end product of the glycolytic pathway, and of [^13^C_2_]glutamate, which is labelled via α-ketoglutarate in the tricarboxylic acid (TCA) cycle (Extended Data Fig. 1b) in GB1 and GB2, were less than that of tumour glucose (Fig. 1d), suggesting that neither had reached isotopic steady state and were therefore a measure of glycolytic and TCA cycle activity, respectively. There was a direct correlation between [^13^C_2_]glutamate signal intensities and the intensities of the TCA cycle intermediates [^13^C_2_]malate, [^13^C_2_]fumarate and [^13^C_2_]succinate (Extended Data Fig. 1c). The signal from ceramide-1-phosphate (752.596 *m*/*z*) confirmed minimal variation in instrument performance during acquisition and uniformity of tissue preparation (Extended Data Fig. 1d). Mass errors on the metabolite ions and the mass isotopologue distributions of all detectable labelled metabolites are given in Supplementary Tables 1 and 2, respectively. The sampling technique rapidly arrested tissue metabolism, as indicated by the ATP/ADP and phosphocreatine PCr/ATP ratios (Fig. 1e,f), which decline rapidly in hypoxic or ischaemic brain tissue^8^. Preservation of the ATP, ADP and PCr concentrations was also demonstrated using ^31^P nuclear magnetic resonance (NMR) measurements (Extended Data Fig. 2a,b). The metabolite ^13^C labelling observed was assumed, therefore, to be similar to that present in vivo. The ATP and PCr signal intensities were similar in the two GB tumours but significantly higher than those in the metastasis (Extended Data Fig. 2c). The concentrations of plasma amino acids and lactate did not change significantly during the infusion protocol. The plasma concentrations of ^13^C-labelled lactate and glutamine, although less than 10% of the unlabelled concentrations, could have contributed to some of the labelled lactate and, via glutamine, the labelled glutamate observed in the tumour sections (Extended Data Fig. 2d and Supplementary Table 3).

### Metabolic profiles of normal cortex and GB tumours

The metastatic adenocarcinoma occupied small, discrete areas surrounded by gliotic and normal-appearing brain parenchyma (Fig. 1g) and therefore provided apparently normal brain tissue for comparative analysis. We segmented the mass spectrometry (MS) images using seven ^13^C-labelled metabolites from glycolysis ([U-^13^C]pyruvate, [U-^13^C]lactate) and the TCA cycle ([^13^C_2_]fumarate, [^13^C_2_]succinate, [^13^C_2_]malate, [^13^C_2_]glutamate and [^13^C_2_]glutamine). This separated the tumour, gliotic and normal-appearing brain parenchyma and agreed with tissue classification performed by a histopathologist (Extended Data Fig. 2e).

Next, we assessed glycolytic activity and TCA cycle activity in the tumours and in normal-appearing brain. [U-^13^C]lactate signals were significantly lower in GB1 than in GB2 and the metastasis, whereas the [^13^C_2_]glutamate signals in GB1 were significantly higher than in the metastasis (Fig. 1h), reflecting higher glycolytic and lower TCA cycle activities in the metastasis. Glutamate labelling in the GB tumours was comparable to that in normal brain.

### Metabolite ^13^C-labelling identifies GB metabolic phenotypes

**Fig. 2:**
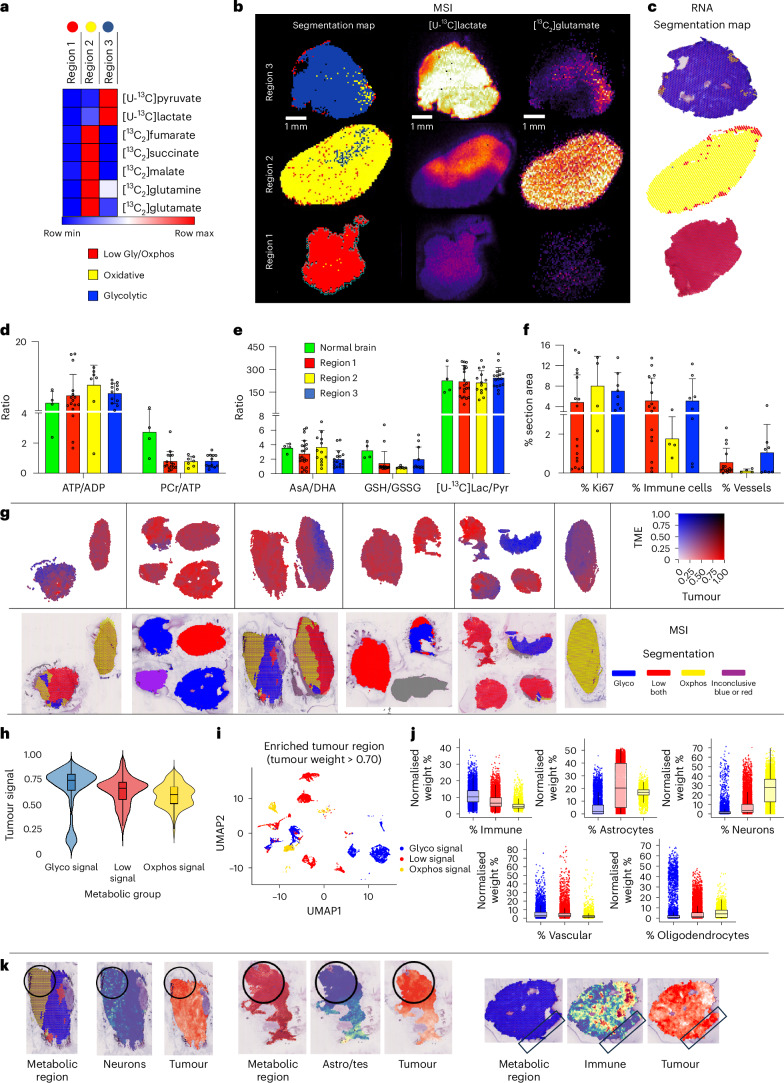
Identification of metabolic phenotypes in GB tumour sections. **a**, Heatmap showing the average intensities of the seven metabolites used for segmentation of the three metabolic regions. **b**, Representative example of three tumour sections segmented into three clusters. The region containing state 3 cells (blue) showed high [U-^13^C]lactate and [U-^13^C]pyruvate labelling and was considered to have a glycolytic phenotype. The region containing state 2 cells (yellow) showed high labelling of [^13^C_2_]glutamate and was considered to have an oxidative phenotype. The region containing state 1 cells (red) showed low labelling of glycolytic and TCA cycle metabolites. **c**, Metabolic segmentation based on spatial RNA sequencing of sections contiguous with those shown in **b**. Blue spots correspond to a glycolytic phenotype, and yellow spots correspond to an oxidative phenotype based on Hallmark gene sets. Red spots correspond to cells with low glycolytic and oxidative gene signatures. **d**, ATP/ADP and PCr/ATP signal intensity ratios (mean; error bars, s.d.) in normal-appearing brain (*n* = 4) and in the three metabolically defined regions (region 1, *n* = 22; region 2, *n* = 14; region 3, *n* = 17); every dot is a unique region. No statistical significance was identified using one-way ANOVA with Tukey’s multiple comparisons test. **e**, Redox status (mean; error bars, s.d.) in normal-appearing brain tissue and GB regions quantified using AsA:DHA, GSH:GSSG and [U-^13^C]lactate/pyruvate ratios (normal brain, *n* = 4; region 1, *n* = 22; region 2, *n* = 14; region 3, *n* = 17). No statistical significance was identified using one-way ANOVA with Tukey’s multiple comparisons test. **f**, Quantification of Ki67^+^ cells, immune cells and blood vessels (mean; error bars, s.d.) in the three metabolically defined regions based on immunohistochemical analysis (region 1, *n* = 17; region 2, *n* = 4; region 3, *n* = 8). No statistical significance was identified using one-way ANOVA with Tukey’s multiple comparisons test. **g**, Spatial co-assignment of deconvolved tumour/TME signals (first row) and MSI labels (second row). **h**, Violin plots showing tumour signal distribution by metabolic phenotype. Box plots display the median (50th percentile) as the central line, with boxes spanning the 25th and 75th percentiles. Whiskers extend to the minimum and maximum values within 1.5× the interquartile range; Glyco, *n* = 5,440; Low, *n* = 8,038; Oxphos, *n* = 4,508. **i**, Spatial uniform manifold approximation and projection (UMAP) plot of tumour-enriched spots from all metabolic phenotypes. **j**, Quantification of TME-deconvolved populations (*x* axis, population; *y* axis, normalized weight per spot for each phenotype). Box plot median and range as in **h**. **k**, Spatial maps showing that areas enriched for tumour cells display all three metabolic states. Highlighted areas show that these metabolic phenotypes arise from cells displaying strong tumour signals rather than from cells of the TME. Source data

**Extended Data Fig. 3:**
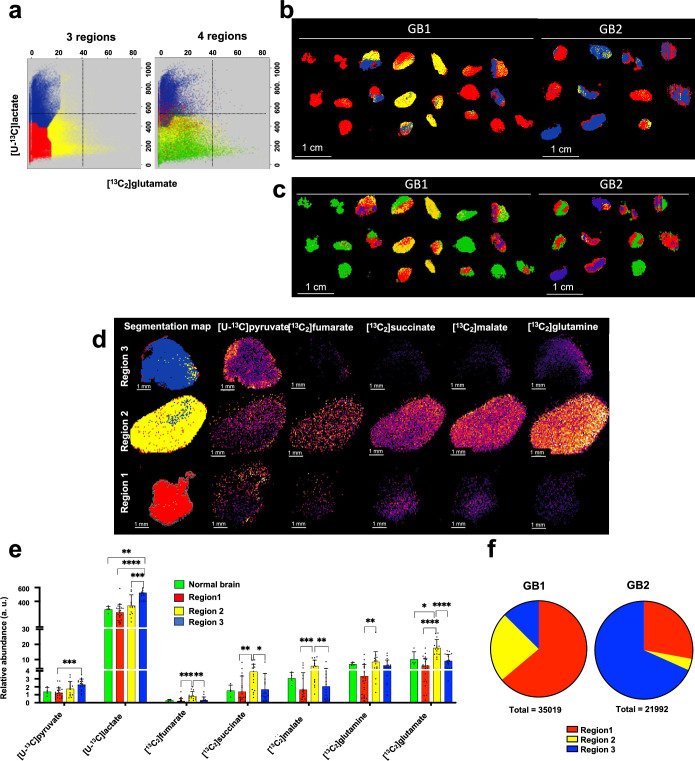
Segmentation of the mass spectrometry images. **a**) Scatter plot of individual pixel intensities in mass spectrometry images of tumour sections from GB1 and GB2. The x-axes shows [^13^C_2_]glutamate and the y-axes [U-^13^C]lactate signal intensities. Colours correspond to the k-means segmentation of pixels into three vs. four groups. **b)** K-means segmentation maps of all the GB1 and GB2 tumour sections assuming three metabolic states. The three metabolic states are represented in red (low activity in glycolysis and TCA cycle), yellow (high TCA cycle activity) and blue (high glycolytic activity). The sections were arranged randomly on the slides for data acquisition. They have been arranged here to match their respective parent tumour in order to assist visualisation. **c)** K-means segmentation maps of all the GB1 and GB2 tumour sections assuming four metabolic states shown in **a**. **d)** Representative GB sections showing the three metabolic states and corresponding signal for ^13^C labelled metabolites in glycolysis and the TCA cycle. **e)** Relative abundance of TCA cycle and glycolytic intermediates in the 3 metabolic states in GB1, GB2 and normal appearing brain (Normal brain n = 4; Region 1 n = 22; Region 2 n = 14; Region 3 n = 17) expressed as mean ± SD. **f)** GB1 had a higher proportion of oxidative regions in the tumor sections (Region 2, yellow) while GB2 had more glycolytic regions (Region 3, blue). The three metabolic phenotypes were present in both patients. Asterisks refer to *P* values obtained from one-way ANOVA test followed by Tukey’s multiple comparisons test or unpaired T-test (*P < 0.05; **P < 0.005; ***P < 0.0005; ****P < 0.00005). Source data

**Extended Data Fig. 4:**
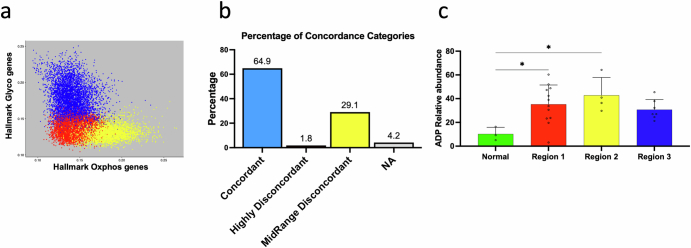
Correlation between the mass spectrometry images and spatial transcriptomics. **a)** Spatial transcriptomic images were segmented using k-means into three metabolic groups, with blue representing highly glycolytic cells (expressing Hallmark glycolytic gene sets), yellow representing oxidative cells (expressing Hallmark oxidative gene sets) and red representing cells low for both glycolysis and oxidative phosphorylation transcripts. **b**) Percent concordance between MSI and RNA derived metabolic states for all tumour pixels. **c)** ADP signal intensities (mean ± SD) were significantly higher in the oxidative regions of GB defined by the MSI data (Normal n = 3; Region 1 n = 12; Region 2 n = 4; Region 3 n = 7). Asterisks refer to *P* values obtained. from one way ANOVA test followed by Tukey’s multiple comparisons test (*P < 0.05; **P < 0.005; ***P < 0.0005; ****P < 0.00005). Source data

We segmented the GB MS images using the same seven ^13^C-labelled metabolites from glycolysis and the TCA cycle. We reasoned that the activity of these two pathways could result in four cellular states; however, the MSI spectra did not contain a high glycolytic, high TCA cycle phenotype, and images were better segmented assuming only three metabolic states (Fig. 2a and Extended Data Fig. 3a): state 3 (high glycolysis, low TCA cycle), state 2 (low glycolysis, high TCA cycle) and state 1 (low activity in both pathways). Segmentation assuming three or four metabolic states showed that these states occupied distinct and spatially extensive regions (Fig. 2b and Extended Data Fig. 3b–e). As observed previously from transcriptomic data^5^, each tumour had a predominant metabolic phenotype, with GB1 containing more of state 2 and GB2 containing more of state 3 (Extended Data Fig. 3f). Nevertheless, regions occupied by one of these three metabolic states co-existed in both tumours, sometimes within a single tumour section. Spatial RNA sequencing of adjacent tumour sections and segmentation using Hallmark oxidative and glycolytic gene sets also found three metabolic states: glycolytic, oxidative and mixed (Extended Data Fig. 4a), which showed a strong correlation with the MSI data (Fig. 2c) and an overall concordance of 65% (Extended Data Fig. 4b).

### Metabolic phenotypes do not correlate with microenvironment

To exclude perfusion and hypoxia as explanations for the observed metabolic heterogeneity, we assessed cellular energy status from measurements of the ATP, ADP and PCr concentrations. All tumour regions had ATP/ADP and PCr/ATP ratios comparable to those in normal brain (Fig. 2d). ADP was more abundant in the more oxidative tumour regions (Extended Data Fig. 4c), consistent with a high intramitochondrial ADP concentration^9^.

The three metabolic states and normal brain showed similar redox status, as assessed from measurements of the ascorbic acid to dehydroascorbic acid (AsA:DHA) and reduced to oxidized glutathione (GSH:GSSG) ratios, reflecting the NADPH/NADP^+^ ratio^10^, and the [U-^13^C]lactate/[U-^13^C]pyruvate ratios, reflecting the NADH/NAD^+^ ratio^11^ (Fig. 2e).

**Extended Data Fig. 5:**
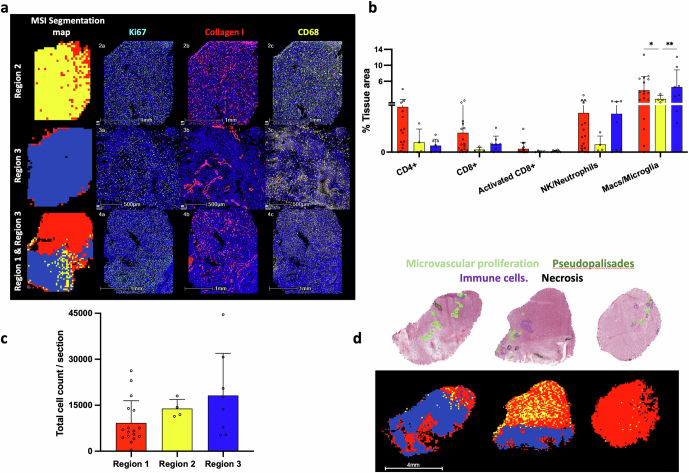
Metabolic phenotypes are not affected by the tumour microenvironment. **a)** Representative IMC images of Ki-67, Collagen I (large vessels), and CD68 (macrophages/microglia) positive cells in regions occupied by cells in one of the three metabolic states in GB tumours. **b**) Quantification of specific immune cells in the regions occupied by one of the three tumour cell metabolic states using IMC (Region 1 n = 17; Region 2 n = 4; Region 3 n = 8; bar is mean ± SD). **c**) Total number of cells quantified using a DNA marker in the metabolically defined regions (Region 1 n = 17; Region 2 n = 4; Region 3 n = 8; bar is mean ± SD). **d)** Representative H&E-stained sections were annotated by a histopathologist for the presence of necrosis, pseudopalisading necrosis, immune cells and microvascular proliferation. The segmented MS images shown below the H&E-stained sections demonstrate a lack of correlation between histological features and metabolic phenotype. Source data

Next, we analysed contiguous sections by imaging mass cytometry (IMC) for the presence of immune cells, blood vessels and proliferating cells. We defined five immune phenotypes: CD3^+^CD45^+^CD4^+^ (helper T cells)^12^; CD3^+^CD45^+^CD8^+^ (cytotoxic T cells)^13^; CD3^+^CD45^+^CD8^+^GZMB^+^ (activated T cells)^14^; CD45^+^GZMB^+^ (natural killer or neutrophils)^15^; and CD68^+^ (macrophages or microglia)^16^. Vascular phenotypes included large vessels (ASMA^+^CollagenI^+^panCK^+^CD31^+^)^17^ and small vessels or capillaries (CD31^+^CollagenI^+^)^18^. Regions occupied by the different metabolic states showed similar numbers of Ki67^+^ cells, immune cell phenotypes and blood vessels (Fig. 2f and Extended Data Fig. 5a,b), with the only significant difference being slightly fewer CD68^+^ cells in the oxidative regions. However, the proportion of immune cells was small, representing less than 10% of the total cell population. Cell density could also not account for the observed metabolic heterogeneity (Extended Data Fig. 5c). Regions of necrosis, pseudopalisading necrosis and microvascular proliferation showed a lack of correlation with metabolically distinct areas (Extended Data Fig. 5d).

**Extended Data Fig. 6:**
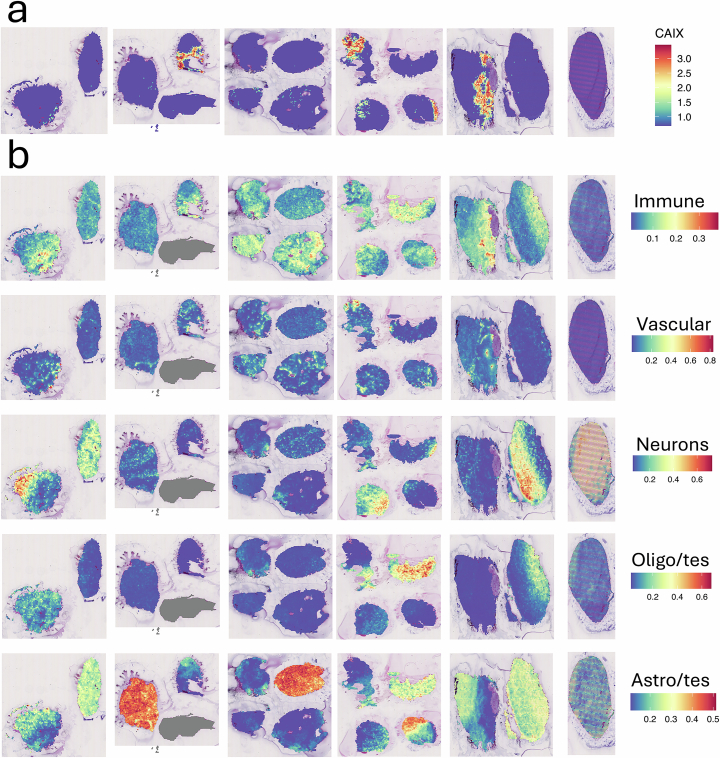
Spatial transcriptomics showed similar numbers of microenviromental cells in tumour regions occupied by the different metabolic states. **a)** Spatial expression of carbonic anhydrase IX (CAIX). **b)** Spatial distribution of RNA expression profiles for immune, vascular, neuron, oligodendrocyte and astrocyte cell populations. Colours indicate the normalised weights and represent the percentage of the deconvolved population present per spot.

Tumour areas identified by a strong malignant signal in the spatial transcriptomics data showed the three metabolic states in spatially coherent areas on co-registered MS images (Fig. 2g–i), whereas regions identified as containing immune cells were predominantly glycolytic and those containing neurons were predominantly oxidative (Fig. 2j). Regions that showed a strong malignant signal and that were largely devoid of TME signals displayed all three metabolic states in the MS images (Fig. 2k). There were no differences in carbonic anhydrase IX (CAIX) transcript levels between the three metabolic states (Extended Data Fig. 6a), suggesting similar levels of hypoxia, or in immune, vascular, neuron, astrocyte and oligodendrocyte cell populations (Extended Data Fig. 6b).

The bioenergetic status and microenvironment of the three metabolic states indicate that differences in metabolic activity are unlikely to have been influenced by differences in tissue perfusion, the presence of necrosis, differences in cell proliferation or the presence of immune cell infiltrates, but rather represent tumour-cell-intrinsic metabolic phenotypes.

### Phenotypes are not associated with vascularization

**Extended Data Fig. 7:**
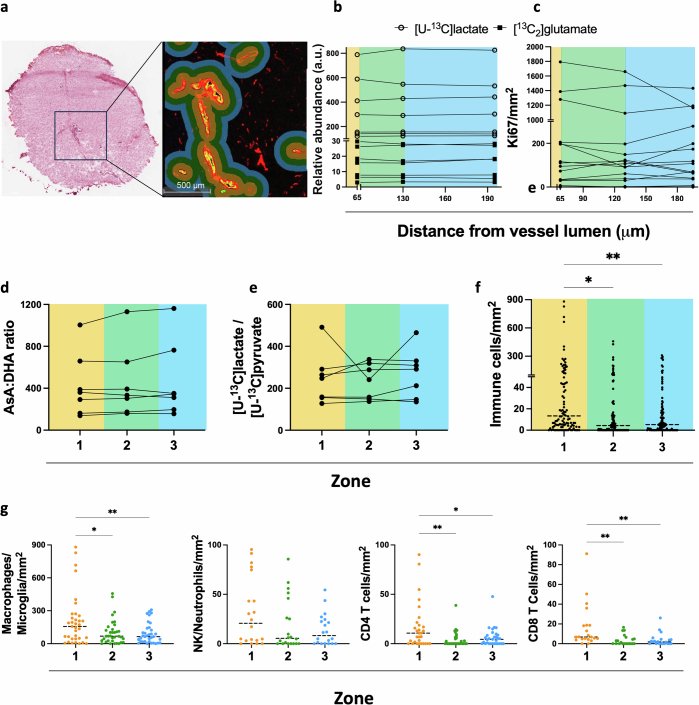
Proximity to the vasculature has no effect on lactate and glutamate labelling. **a)** *Left:* Representative H&E-stained GB section and (*right*) IMC of a contiguous section with double positive *Collagen I+* and *αSMA* vessels. Three infiltration bands (yellow, green, blue), 65 μm wide, radiating from the vessel wall are shown. **b)** Relative abundance of [U-^13^C]lactate and [^13^C_2_]glutamate at the specified distances from the vessel wall. The colours correspond to the bands shown around each vessel in **a)**. **c**) Number of Ki67 positive nuclei/mm^2^ in each infiltration band. The colours correspond to the infiltration bands around each vessel in **a)**. **d)** AsA:DHA and **e)** [U-^13^C]lactate/[U-^13^C]pyruvate ratios at 65 μm, 130 μm and 195 μm from the vessel wall. **f)** Total number of immune cells (n = 118 infiltration bands) and **g)** the number of specified immune cell subtypes within each infiltration band (Macs/microglia bands n = 35; NK/neutrophil bands n = 20; CD4+ n = 31; CD8+ n = 21). There were significantly more immune cells in the band closest to the vessel. Data are mean ± SD, where every dot is a tumour section or a region of a tumour section identified by k-means segmentation. Asterisks refer to *P* values obtained from one-way ANOVA test followed by Tukey’s multiple comparisons test or unpaired T-test (*P < 0.05; **P < 0.005). Source data

Despite there being no significant correlation between metabolic phenotype and blood vessel density, we nevertheless investigated a possible relationship between metabolic phenotype and proximity to the vasculature. We selected large vessels (Collagen I^+^ and αSMA^+^) (Extended Data Fig. 7a), co-registered these with contiguous MSI sections and measured ^13^C-labelled glycolytic and TCA cycle metabolites at 65 μm intervals from the blood vessel lumen. Regardless of distance, there were no differences in lactate and glutamate labelling (Extended Data Fig. 7b), the number of proliferating cells, as indicated by Ki67^+^ staining (Extended Data Fig. 7c), the NADPH/NADP^+^ ratio (AsA:DHA ratio) or the NADH/NAD^+^ ratio ([U-^13^C]lactate/[U-^13^C]pyruvate ratio) (Extended Data Fig. 7d,e). However, there was a decrease in the number of immune cells with increasing distance from the vessel wall (Extended Data Fig. 7f,g).

### Metabolic phenotypes are preserved in primary neurospheres

**Fig. 3:**
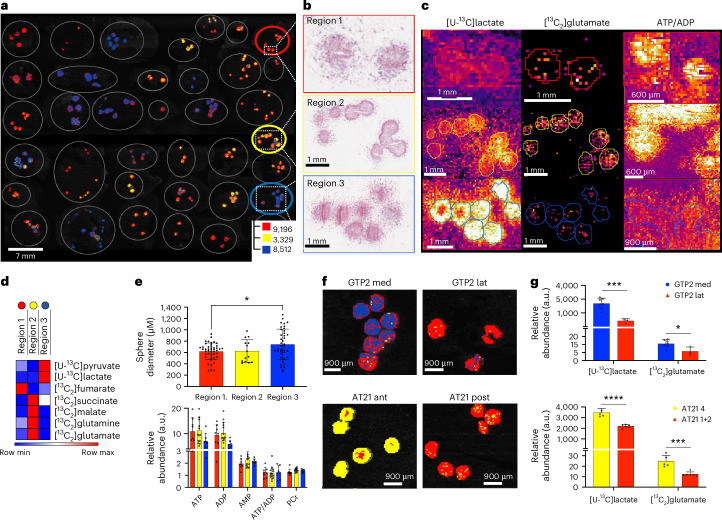
Primary neurospheres recapitulate metabolic phenotypes in patients with GB. **a**, *k*-means clustering map of 30 cell lines grown as neurospheres in Matrigel domes (outlined in grey) using the seven ^13^C-labelled metabolites from glycolysis and the TCA cycle. Red corresponds to metabolic state 1 (low glycolytic, low TCA cycle activity), yellow corresponds to metabolic state 2 (high TCA cycle activity) and blue represents metabolic state 3 (high glycolytic activity). **b**, Representative H&E-stained sections of spheres displaying one of the three metabolic states. Red, yellow and blue outlines correspond to the sphere domes in **a**. **c**, Signal intensity maps for [U-^13^C]lactate, [^13^C_2_]glutamate and the ATP/ADP ratio in spheres shown in **b**. **d**, Heatmap showing the average signal intensities of the seven metabolites used for segmentation of the three metabolic states. **e**, Top: sphere diameter for each metabolic phenotype. Each point represents a single sphere. State 3 (glycolytic phenotype) had a higher average sphere diameter than state 1 (low glycolytic, low TCA cycle activity) (*P* = 0.027). Bottom: ATP, ADP, AMP and PCr signal intensities in neurospheres with different metabolic phenotypes. **f**, *k*-means segmentation of MS images of spheres derived from multi-regional tumour sampling (GTP2 and AT21). These results are part of the *k-*means analysis performed on all 30 neurosphere lines. **g**, Signal intensities of [U-^13^C]lactate and [^13^C_2_]glutamate in the indicated neurospheres (****P < 0.0001). The colour of the bar corresponds to the metabolic phenotype. Data are means; error bars, s.d.; each dot represents a single neurosphere. Asterisks refer to *P* values obtained from one-way ANOVA followed by Tukey’s multiple comparisons test or unpaired *t*-test (*P < 0.05, ***P < 0.0005, ****P < 0.00005). Source data

**Extended Data Fig. 8:**
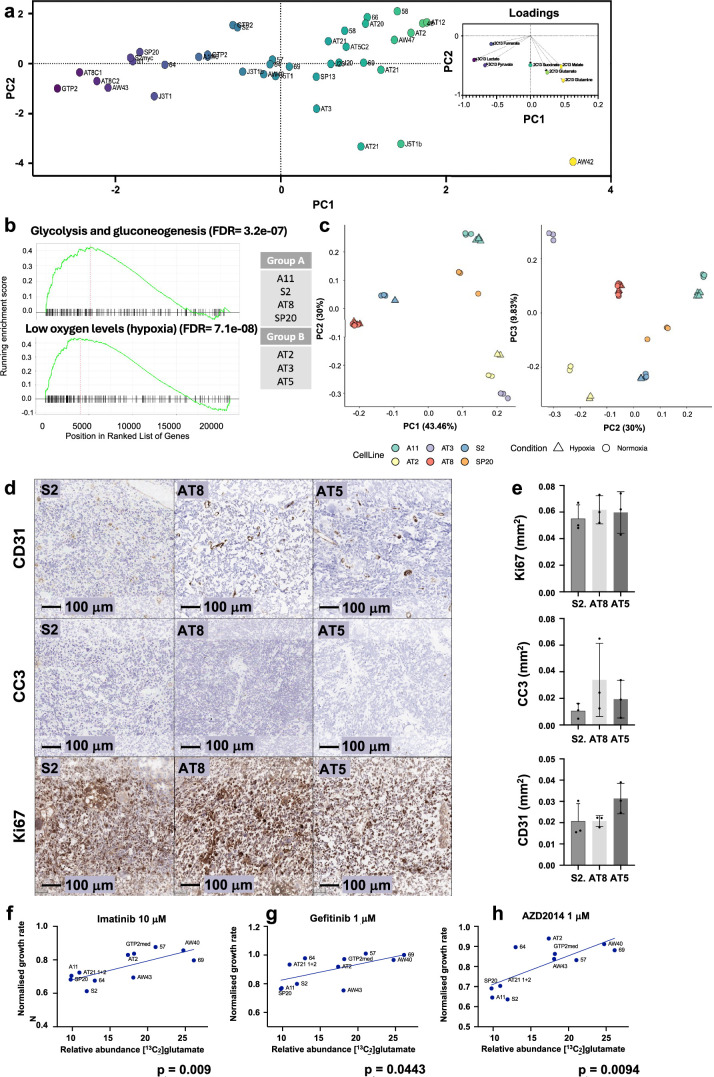
Characterisation of neurosphere and xenograft metabolic phenotypes. **a**) PCA plot using MSI signal intensities of ^13^C-labelled pyruvate, lactate, succinate, malate, fumarate, glutamate and glutamine. The loadings plot (inset) shows that the oxidative spheres separate to the right of PC1 and the glycolytic spheres to the left. Although the MSI data were segmented into 3 metabolic phenotypes, the relative activities of the glycolytic and oxidative pathways are a continuum. **b**) Neurosphere RNA sequencing DGSEA analysis showed upregulation of hallmark genes in glycolysis and hypoxia in the more glycolytic spheres, as determined by MSI (labelled here as group A). **c**) Neurospheres grown in normoxia and hypoxia (0.5% O_2_). PCA plot of RNA sequencing data showing separation of neurospheres into three groups. Hypoxia did not shift the position of these groups on PC1 and PC2 significantly. **d)** Contiguous sections from S2, AT8 and AT5 xenografts stained for Ki-67, CC3 and CD31. **e)** Ki67, CC3 and CD31 signals normalized to tissue area (mm^2^) were not significantly different between the three tumour models (n = 3 biological replicates per tumour model; data presented as mean ± SD). **f, g, h)** Correlation of glutamate labelling in the primary cell lines with response to Imatinib (R^2^ = .55, F(1, 9) = 11.0, p = 0.009) **(f)**, Gefitinib (R^2^ = .38, F(1, 9) = 5.46, p = 0.0443) **(g)** and AZD2014 (R^2^ = .55, F(1, 9) = 10.83, p = 0.0094) **(h)** treatment. The y-axis shows cell line response to drug normalised to control, where 1.0 indicates unsuppressed growth in the presence of the drug and values less than 1.0 indicate reduced cell growth relative to control. Source data

To confirm that the metabolic phenotypes are tumour-cell-intrinsic and not a consequence of differences in the TME, we derived 30 primary cell lines from 26 patients with GB (two patients had two cell lines derived from multi-regional tumour sampling). These were grown as neurospheres with [U-^13^C]glucose before snap-freezing and sectioning for MSI analysis (Fig. 3a–c). Three-dimensional culture has been shown to more closely approximate the behaviour of the primary tumour^19^. The MS images were segmented using the same *k*-means clustering as for the human data (Fig. 3d). The neurospheres derived from each cell line showed distinct metabolic states (Fig. 3a). Neurosphere diameter was similar for all three metabolic phenotypes, which showed similar AMP, ADP, ATP and PCr signal intensities and ATP/ADP ratios (Fig. 3e). Although the segmentation allowed us to group the spheres and the cells from which they were derived into distinct metabolic phenotypes, these nevertheless represent a continuum of glycolytic and TCA cycle activities (Extended Data Fig. 8a). RNA sequencing showed that spheres with a glycolytic phenotype had an upregulation of glycolytic genes and those regulated by hypoxia (Extended Data Fig. 8b). However, there was no difference in the oxidative or TCA cycle gene expression profiles.

Next, we looked at neurospheres derived from multi-regional sampling of the same tumour to determine whether the neurospheres captured the regional metabolic heterogeneity observed in the tumour samples. GTP2 Med (medial tumour) and GTP2 Lat (lateral tumour) formed similar-sized neurospheres but showed different metabolic phenotypes, with GTP2 Med being more glycolytic and clustering into state 3 and GTP2 Lat clustering into state 1. Similarly, AT21 Ant (anterior) and AT21 Post (posterior), from another patient, displayed an oxidative and mixed metabolic phenotype, respectively (Fig. 3f,g). Therefore, metabolic heterogeneity observed in vivo was retained following tissue dissociation and growth outside of the native TME.

To test the robustness of the tumour-cell-intrinsic metabolic phenotype, we grew a subset of neurospheres, representative of the highly glycolytic to the more oxidative phenotypes, under normoxic and hypoxic conditions (0.5% O_2_) and compared their transcriptomes. Despite prolonged exposure to hypoxia (160 h), there was minimal change in the transcriptomes (Extended Data Fig. 8c). Gene set enrichment analysis of the PC1 loadings showed E2F and MYC targets and mTORC1 pathway genes in the top ten under both normoxic and hypoxic conditions, with hypoxia and glycolysis genes under normoxic conditions and oxidative phosphorylation, and epithelial mesenchymal transition genes under hypoxic conditions (Supplementary Table 4).

### Neurosphere phenotypes were reproduced in rat brain

**Fig. 4:**
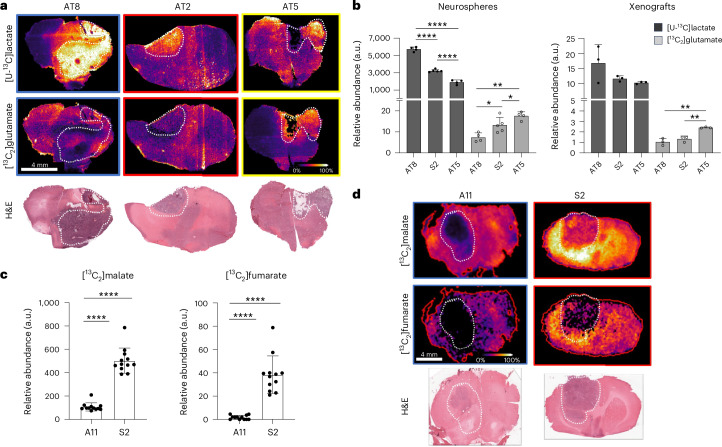
Neurospheres retain their metabolic signatures as orthotopically implanted xenografts, and these signatures correlate with drug response. **a**, Representative MSI sections from rat brains implanted with AT8, S2 and AT5 (*n* = 3 independent tumours per model). Relative signal intensities for [U-^13^C]lactate (top) and [^13^C_2_]glutamate (bottom); H&E-staining of the corresponding sections. **b**, The relative signal intensities of [U-^13^C]lactate (****P < 0.0001) and [^13^C_2_]glutamate (AT8 vs AT5, *P* = 0.0012; S2 vs AT5, *P* = 0.0406; AT8 vs AT5, *P* = 0.0012) in neurospheres (AT8, *n* = 4; S2, *n* = 5; AT5, *n* = 4) and the respective xenografts (AT8, *n* = 3; S2, *n* = 3; AT5, *n* = 3) expressed as mean values; error bars, s.d. ([^13^C_2_]glutamate AT8 vs AT5, *P* = 0.0015; S2 vs AT5, *P* = 0.0049). *P < 0.05; **P < 0.005. **c**, Relative abundance (mean values; error bars, s.d.) of labelled malate and fumarate in sections of A11 and S2 xenografts. **d**, Top: labelled malate and fumarate signal intensities in MS images of the brains of rats implanted with A11 (*n* = 12) and S2 (*n* = 12) xenografts. Bottom: H&E-staining of the corresponding sections. Asterisks refer to *P* values obtained from one-way ANOVA followed by Tukey’s multiple comparisons test or unpaired *t*-test (****P < 0.0001). Source data

To further test the cell-intrinsic nature of the metabolic phenotypes, we implanted A11, AT8, S2 and AT5 into the brains of athymic rats and found that the cells retained the metabolic phenotypes that were observed when they were grown as neurospheres. AT8 and S2 formed more glycolytic xenografts, as demonstrated by higher [U-^13^C]lactate labelling, whereas AT5 xenografts were more oxidative with higher abundance of [^13^C_2_]glutamate (Fig. 4a,b). There were no differences in cell proliferation (Ki67 staining), cell death (CC3 staining) or vascularization (CD3 staining) between these three models, which paralleled the finding in the human data that proliferation rate and vascularization were not responsible for the differences in the observed metabolic phenotypes (Extended Data Fig. 8d,e). We have shown previously that S2 cells are more sensitive than A11 cells to irradiation (referred to as GB1 and GB4, respectively, in the earlier publication^20^) and that S2 xenografts are more sensitive than A11 xenografts to treatment with temozolomide plus irradiation^21^. Although when evaluated alongside all the other neurospheres, A11 and S2 appeared similarly glycolytic (Extended Data Fig. 8a), S2 xenografts showed greater TCA cycle activity than A11 xenografts (Fig. 4c,d). We have shown previously that A11 cells show higher glycolytic activity and S2 cells higher oxygen consumption and lower glycolytic activity, and demonstrated this in the corresponding xenografts using deuterium magnetic resonance spectroscopy and spectroscopic imaging measurements of deuterium-labelled glucose metabolism in vivo^22^. Treatment of cells with AZD2014 (mTOR1 and mTOR2 inhibitor)^23^, imatinib (PDGFR; tyrosine kinase inhibitor)^24^ and gefitinib (EGFR; tyrosine kinase inhibitor)^25^ showed that cell viability was correlated with metabolic phenotype, where oxidative cells were more drug resistant (Extended Data Fig. 8f–h).

### Phenotypes showed signatures in other pathways

**Extended Data Fig. 9:**
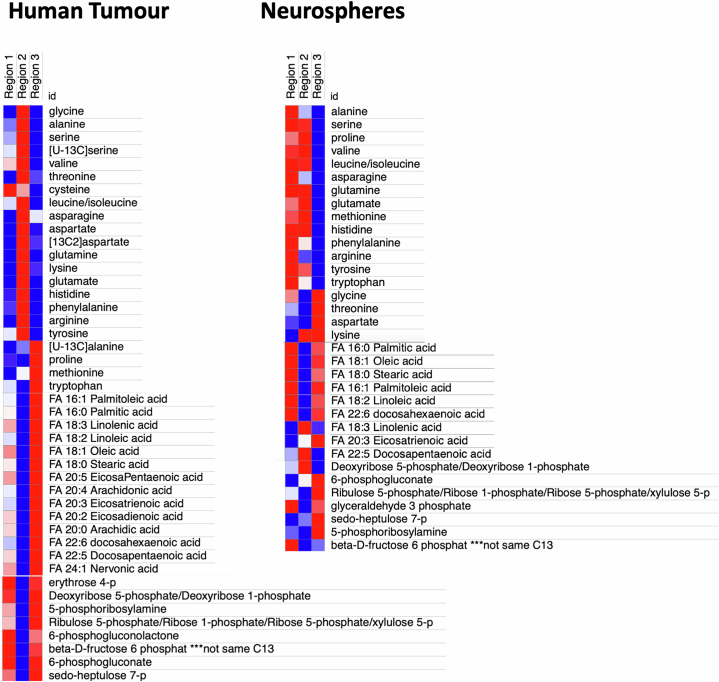
The metabolic phenotypes show signatures in other pathways. Concentrations of amino acids are higher in Region 2 (oxidative) in both human GB sections and neurospheres. The concentrations of fatty acids and PPP intermediates are higher in Region 1 and Region 3 (which are both more glycolytic than Region 2). There are fewer metabolites shown in the neurosphere data because the DESI-Orbitrap data was acquired over a greater m/z range (70-1000 m/z) while the human data was acquired over a narrower range (70-280 m/z). This reduces the sensitivity of detection in neurospheres, particularly of the less abundant metabolites.

The concentrations of unlabelled serine, threonine, glutamine and glutamate were significantly higher in oxidative regions in the patient tumour sections (ANOVA, Tukey’s *P* < 0.05), and in the neurospheres, the concentrations of unlabelled leucine/isoleucine, glutamine, glutamate, histidine and phenylalanine were significantly higher (ANOVA, Tukey’s *P* < 0.005) (Extended Data Fig. 9). The oxidative phenotype in GB has been associated with increased fatty acid oxidation^5^, and we observed that the concentrations of unlabelled fatty acids were higher in the more glycolytic regions in both the human and neurosphere data, as were intermediates in the pentose phosphate pathway.

## Discussion

The extent to which tumour metabolism is driven by cell-intrinsic mechanisms or microenvironmental pressures is an open question in tumour biology^6^. To address this question, we measured the metabolic activity of GB within its native microenvironment using MS imaging of isotope labelling in rapidly quenched tissue from patients with GB. A similar approach has been used to map heterogeneity in fatty acid synthesis in gliomas implanted orthotopically in mice^26^, to map metabolic activities in kidney and brain in mice^27^ and to image glycolytic activity in a lung metastasis model^28^.

Using a targeted approach, we identified distinct glycolytic and oxidative metabolic phenotypes. Although recent reports have identified metabolic phenotypes from a transcriptomic analysis^5^, we describe here the classification of metabolic phenotypes based on measurements of metabolic activity in patient tumours in vivo at relatively high spatial resolution (65 μm). The metabolic phenotypes occupied distinct territories that did not show significant differences in the cellular composition of their microenvironments, including immune cell infiltration, proliferative index and vascularization. The presence of these metabolic phenotypes in distinct territories and their cellular composition was confirmed by spatial transcriptomics. Importantly, we have shown that these tumour-cell-intrinsic metabolic phenotypes can be independent of the TME. Previous studies have also shown that tumour subtype-specific protein^29^ and gene expression profiles^30^ can be independent of the tumour niche.

We observed no change in lactate and glutamate labelling with distance from the blood vessels. This appears to be inconsistent with a study in an orthotopically implanted glioma cell line model (U87MG) in immunocompromised mice that showed high mitochondrial activity in cells adjacent to vessels and increased expression of hypoxia-related genes at increasing distance from the vessels. However, there were no significant increases in lactate concentration or differences in α-ketoglutarate concentrations in the cells with distance from the vessels in this study, although these concentrations may have been affected by the flow cytometric method used to sort the cells^31^. An MSI study on an orthotopically implanted syngeneic murine model of isocitrate dehydrogenase 1 mutant GB in animals fed for 48 h with [U-^13^C]glucose showed a remarkable degree of metabolic homogeneity in the tumours^32^, in contrast to what was observed here in tumours from patients with GB, emphasising the significance of the observed metabolic phenotypes.

The cell-intrinsic nature of the metabolic phenotypes was confirmed using neurospheres grown in vitro, which reproduced the metabolic phenotypes observed in the patients and were preserved following their orthotopic implantation in rats. Exposing the neurospheres to chronic hypoxia did not lead to significant changes in the neurosphere transcriptomes, demonstrating the robustness of these phenotypes and further underlining their independence from the TME. This is in contrast to a similar study in patients with lung cancer who were infused with [U-^13^C]glucose, which concluded that given the observed differences in glucose oxidation in the TCA cycle in well-perfused versus poorly perfused regions, tumour perfusion in this case overrides tumour-cell-intrinsic metabolic phenotypes^33^.

A recent study in mouse models of leukaemia, pancreatic, lung and colon cancer showed that these tumours suppress TCA cycle activity relative to normal tissue. By contrast, the glutamate labelling observed here in the GB tumours was similar to that in normal-appearing brain. Similar observations of substantial glucose oxidation in the TCA cycle have been made previously in human lung tumours^33^ and in non-imaging studies of patients with GB who were infused with [U-^13^C]glucose^7^.

Metabolic phenotypes with distinct therapeutic vulnerabilities have been identified in several cancers^34–38^, including GB^5^, in which GB cells with an oxidative phenotype were shown to be more sensitive to inhibitors of mitochondrial complex I and to radiation treatment. We have shown previously that S2 cells are more sensitive to irradiation in vitro^20^ and in vivo^21^ and have shown here, and previously, that they have a more oxidative phenotype with higher TCA cycle activity than A11 cells, which show a more glycolytic phenotype. Previous RNA sequencing studies have shown that A11 has a mesenchymal phenotype, whereas S2 cells have a neural progenitor cell-like phenotype^39^, consistent with a previously identified association between the mesenchymal and glycolytic phenotypes^5^.

Cells displaying these distinct metabolic phenotypes occupy territories that are sufficiently large to be imaged clinically using techniques such as hyperpolarized ^13^C magnetic resonance imaging (MRI)^40^ and deuterium metabolic imaging^41^. These metabolic phenotypes show a correlation with treatment responsiveness, suggesting that a personalized therapy approach may be possible in which metabolic imaging and phenotyping could be used to guide subsequent treatment selection.

We have demonstrated here high-resolution imaging of isotope labelling of cellular metabolites in a human tumour in vivo. In conjunction with studies on patient-derived neurospheres and orthotopically implanted xenografts, we have demonstrated the presence of different metabolic phenotypes within GB that are tumour-cell-intrinsic and largely independent of the TME.

## Methods

### Patient samples and ethical approval

Three male patients from Addenbrooke’s Hospital, Cambridge, were infused with [U-^13^C]glucose. The selection criteria included first clinical presentation, MRI consistent with GB and no significant co-morbidities.

Following induction of anaesthesia, a pyrogen-free 5% solution of [U-^13^C]glucose in sterile saline (Merck) was administered as a bolus of 8 g over 10 min followed by 8 g h^−1^ continuous infusion, as described previously for patients with GB^7^ and several other tumour types^42^. Arterial blood was collected by a peripheral arterial line before bolus administration and then every 15 min following the start of infusion and at 15 min after the end of the infusion. Tumour sampling was guided by intra-operative Stealth navigation^43–45^ and assessment of 5-ALA fluorescence. Tumours were sampled between 90 and 150 min^7^. A pituitary ronguer was used to transfer tumour samples directly into liquid nitrogen. The freezing time was <5 s between tissue devascularization and immersion in liquid nitrogen.

The study was approved by the Central Cambridge Research Ethics Committee and was compliant with the Health Research Authority. The study adhered to the principles of the Declaration of Helsinki and the Guidelines for Good Clinical Practice. Participants did not receive financial compensation and gave informed consent.

### Tissue preparation

Frozen tumour samples were embedded in a hydroxypropyl methylcellulose/polyvinylpyrrolidone hydrogel^46^, and 10 µm-thick cryo-sections were obtained. Sections were thaw-mounted onto Superfrost microscope slides for desorption electrospray ionization (DESI) and IMC experiments (Thermo Scientific), while sections prepared for MALDI-MSI were thaw-mounted onto conductive ITO-coated slides (Bruker Daltonik). The sections were dried immediately and sealed in vacuum pouches for storage at −80 °C. Human tumour samples were treated with UV-C light before MSI analysis to minimize aerosolization of potential pathogens. Decontamination was performed in a sensor-controlled UV chamber (Opsytec Dr. Gröbel) at 250 mJ cm^−2^.

### MSI

DESI-MSI analysis was performed on a Q-Exactive mass spectrometer (Thermo Scientific) equipped with an automated 2D-DESI ion source (Prosolia). The spectrometer was used with a home-made Swagelok DESI sprayer and a mixture of 95% methanol, 5% water delivered at a flow rate of 1.5 µl min^−1^ and nebulized with nitrogen at a backpressure of 6 bar. Human tumour samples were analysed in the mass range from 70 to 280 *m*/*z* with a mass resolution of 140,000 at 200 *m*/*z*. The injection time was set at 500 ms, and data were acquired with a pixel size of 65 µm. Following analysis, sections were stained with H&E and co-registered with the DESI-MS images. The resulting .raw files were converted into .mzML files using ProteoWizard msConvert (v.3.0.4043) with the built-in peak picking algorithm^47^ and subsequently compiled to an .imzML file (imzML converter, v.1.3)^48^. All subsequent data processing was performed in SCiLS Lab (v.2022b, Bruker Daltonik).

MALDI-MSI analysis was performed using a RapifleX Tissuetyper instrument (Bruker Daltonik) operated in negative ion detection mode. 9-Aminoacridine, prepared in an 80:20 methanol-to-water ratio, was used as a matrix and spray-deposited using an automated spray system (M3-Sprayer, HTX technologies). Mass spectra were acquired from 180 to 1,000 *m*/*z* with a pixel size of 40 µm. A total of 350 laser shots were summed up per pixel. For all experiments, the laser was operated with a repetition rate of 10 kHz. Raw data were directly uploaded and processed with SCiLS Lab (v.2022b) software.

DESI and MALDI data and images were normalized to the total ion current to compensate for signal variation during the course of the experiments, and acquisition parameters and data processing were identical for human tissue, neurospheres embedded in Matrigel and xenograft sections.

### ^31^P NMR spectroscopy of tumour extracts

Snap-frozen tumour samples were homogenized with 5 µl mg^−1^ of 2 M perchloric acid. The extract was centrifuged at 13,000*g* for 15 min, and the pH of the supernatant was adjusted to 7.0 using 2 M KOH. Extracts were lyophilized and dissolved in 550 µl deuterium oxide containing methylenediphosphonic acid at 100 nmol g^−1^ tissue, which was added as a chemical shift and intensity standard. Proton-decoupled ^31^P NMR spectra were acquired with more than 8,000 repetitions into 32,768 data points with an 11 µs 90° pulse, a repetition time of 7.2 s and a spectral width of 57 ppm (14,006 Hz).

### Liquid chromatography–MS plasma analysis

Arterial blood collected during intra-operative infusion was centrifuged at 2,000*g* and 4 °C for 20 min to collect the plasma, which was snap-frozen and stored at −80 °C. Samples were thawed on wet ice and aliquots diluted 50-fold with cold methanol:acetonitrile:water (50:30:20) in chilled tubes, vortexed for 10 min and centrifuged at 21,100*g* for 10 min at 4 °C, and then the supernatants stored at −80 °C. On the day of analysis, supernatants were centrifuged and aliquots transferred to a 96-well plate for analysis by hydrophilic interaction liquid chromatography (HILIC) high-resolution mass spectrometry (HRMS).

The HILIC–HRMS system consisted of a Shimadzu Nexera X2 UHPLC and Sciex 6600 Triple TOF mass spectrometer, using a SeQuant ZIC-pHILIC 5 µm 150 × 2.1 mm column (with a ZIC-pHILIC 20 × 2.1 mm guard column) at 45 °C. The liquid chromatography gradient started at 80% acetonitrile and 20% 20 mM ammonium carbonate (pH 9.4), changing to 20% acetonitrile over 17 min at 200 µl min^−1^, with a further 11.5 min of column re-equilibration. Each sample was injected twice for analysis in both positive and negative electrospray ionization with a full-scan *m*/*z* range of 75–1,000.

Data were acquired using Sciex Analyst TF and processed using Sciex MultiQuant software. Extracted ion chromatograms were generated from the theoretical *m*/*z* ± 20 ppm. Peak integration was reviewed manually, and the peak area of each metabolite and isotope was exported.

### Neurosphere cell line derivation and MSI preparation

Primary human cell lines were derived at Addenbrooke’s Hospital, Cambridge, UK, as described previously^49^. Tissue collection was approved by a Regional Ethics Committee (REC18/EE/0283) and was compliant with the UK Human Tissue Act 2004. Resected tissue samples were washed with Hanks’ balanced salt solution and minced using sterile razor blades, followed by digestion with Accutase (Sigma-Aldrich) for 60 min at 37 °C. Single cells were isolated by filtration through a 40 µM filter (Falcon). Cells were centrifuged at 350*g*, 21 °C, and the pellet was incubated with 2–3 ml of Red Blood Cell Lysis Buffer (Sigma-Aldrich) for 5 min at room temperature (21 °C). Following centrifugation, cells were seeded at a density of 15,000 cells per cm^2^ in serum-free Neurobasal A medium (Gibco) supplemented with B27, N2, EGF and FGF growth factors (Sigma-Aldrich). Cells were allowed to form aggregates, and the medium was changed 3 days post derivation^50^.

For neurosphere formation, cells were seeded in Ultra Low Attachment 96-well plates (Corning) at a density of 10,000 cells per well. Sphere diameter was monitored using an IncuCyte microscope (Sartorius). Spheres were embedded in 150 μl Matrigel (Corning) domes in 24-well plates (Corning). The domes were covered with 2 ml of fresh Neurobasal medium and grown for 24 h. The domes were then washed three times with PBS before 2 ml of fresh glucose-free Neurobasal medium supplemented with 25 mM [U-^13^C]glucose was added. Following incubation for 24 h, the medium was removed and the domes were washed three times with ice-cold PBS and then snap-frozen in liquid nitrogen. The domes were sectioned to a thickness of 10 µm and analysed using DESI-MSI and MALDI-MSI, as described above.

### Drug treatment of primary cells

Black, clear-bottomed 96-well plates (Corning) pre-coated with ECM (Merck) were seeded at 5,000 cells per well and incubated at 37 °C in 200 μl of Neurobasal medium supplemented with growth factors, as described above. The medium was then replaced with either fresh medium or medium containing the following drug concentrations: AZD2014, 1 μM; gefitinib, 1 μM; imatinib, 10 μM. The cells were incubated for 72 h before re-imaging using the IncuCyte microscope, and cell numbers were determined using the Sartorius cell confluency module. At the end of the incubation, the medium was aspirated, and 100 μl of PBS containing 3 μM propidium iodide was added to each well to measure necrotic cell death. The plates were imaged using the IncuCyte 10× cell-by-cell module using the red channel. The experiment was repeated three times with six technical replicates for each drug treatment.

### RNA sequencing of neurospheres

Neurospheres in Matrigel domes were grown at 37 °C, with one plate incubated in atmospheric O_2_ and the other grown in 0.5% O_2_, 0.5 Pa (Avatar, XCellbio). Medium was refreshed every 48 h, and on day 10 it was removed, the plates placed on ice and the wells washed with 1 ml of ice-cold PBS, followed by the addition of 1 ml Corning Cell Recovery Solution. The plates were incubated at 4 °C for 1 h and then washed four times with ice-cold PBS. RNA was isolated from cell pellets using a QIAshredder spin column (Qiagen) and AllPrep DNA/RNA Mini Kit (Qiagen), quantified using a Qubit fluorometer (Thermo Fisher Scientific) and quality tested using an Agilent 4200 TapeStation. For library preparation, the Illumina TruSeq Stranded mRNA Kit was used, and single-read sequencing was performed on a HiSeq 4000 machine (Illumina). Quality control of raw sequence data was carried out using FastQC (v.0.11.8). Some reads were trimmed to remove adaptor content using Trimmomatic (v.0.39)^51^. Reads were aligned to GRCh38 Ensembl release 102 using STAR (v.2.7.7a)^52^ and alignment quality control was carried out using Picard tools (v.2.25.1). Quantification was carried out using Salmon (v.1.6.0) against a reference transcriptome for the same genome release. Differential gene expression analysis was carried out in R (v.4.2.2) using the DESeq2 package (v.1.38.3)^53^ with default parameters. Multiple testing correction of *P* values was carried out using the Benjamini–Hochberg method^54^. Genes were determined to be differentially expressed at an adjusted *P* value of 0.05. Gene set enrichment analysis was carried out using clusterProfiler (v.4.6.0)^55^.

### Patient-derived xenografts and [U-^13^C]glucose infusion

Experiments were performed under the authority of a Home Office project licence (PP5634271) and approved by an Animal Welfare and Ethical Review Body at the Cancer Research UK (CRUK) Cambridge Institute, University of Cambridge. Athymic, female nude rats that were at least 9 weeks old were implanted orthotopically with the primary GB lines at passage 10. Animals were anaesthetized using 2% isoflurane (Isoflo, Abbott Laboratories) in O_2_/air (25/75%, vol/vol, 2 l min^−1^) with 5 mg kg^−1^ Carpofen (Zoetis) and 0.3 mg ml^−1^ buprenorphine hydrochloride (Alstoe) subcutaneous analgesia. Body temperature was maintained using a heating pad. A stereotactic surgical frame (Kopf) was used to secure the animal’s head. A midline incision was made followed by a 1 mm burr hole anterior and to the right of the bregma. A total of 1 × 10^6^ cells were injected in 5 µl of Neurobasal medium at a depth of 4 mm. The burr hole was closed with bone wax (Ethicon) and skin with 6/0 vicryl (Ethicon). Tumour growth was monitored using T_2_-weighted MRI. Specifically, animals were anaesthetized using 2% isoflurane (Isoflo, Abbott Laboratories) in O_2_/air (25/75%, vol/vol, 2 l min^−1^) and placed supine inside a 7T magnet (Agilent), and T_2_-weighted MRI was used to monitor tumour growth. A 72 mm ^1^H volume coil was placed around the animal’s head, and breathing rate and temperature were monitored with a small animal instruments monitoring system (SAII). Axial ^1^H T_2_-weighted images were acquired using a fast spin-echo sequence with an echo time of 50 ms, pulse repetition time of 1,500 ms, flip angle of 60–90° and a slice thickness of 2.0 mm, field-of-view of 40 mm × 40 mm and 128 × 128 or 256 × 256 data points.

Three animals per cell line were administered with [U-^13^C]glucose as a bolus at 0.4 mg g^−1^, followed by continuous infusion of 0.012 mg g^−1^ min^−1^ at 300 µl h^−1^ for 120 min (ref. ^56^). The brains were snap-frozen in liquid nitrogen, cryo-sectioned at a thickness of 10 µm and analysed with DESI-MSI and MALDI-MSI, as described above.

### Spatial transcriptomic data acquisition and analysis

The 10× Genomics Visium platform was used and analysed with the Space Ranger pipeline. Downstream analyses were conducted in R using the Seurat package^57^. Samples were processed individually using the SCTransform() function. Spots were filtered based on standard quality control thresholds (for example, mitochondrial gene percentage of >20%, nCount_Spatial of <1,000 and nFeature_Spatial of <1,000). Data were re-corrected across samples using PrepSCTFindMarkers() for joint numerical analyses. Gene set scoring was performed using the Hallmark gene sets and the UCELL package^58^, excluding mitochondrial genes from the oxidative phosphorylation score calculations. To annotate spatial spots, genes from Hallmark gene sets of interest were combined and subjected to *k*-means clustering (*k* = 3).

To annotate TME spots, we performed two deconvolution steps with robust cell type decomposition^59^, using previously published reference cell annotations^60^. First, a balanced normal reference was sampled from the non-neoplastic cells, combined with the neoplastic cells and used as input for robust cell type decomposition. The assignment was further confirmed by histological evaluation. To label TME niches, we further deconvolved TME signals into oligodendrocytes, astrocytes, vascular cells, immune cells (macrophages) and neurons. Given that each spatial spot contained multiple cells, we normalized the deconvolution weights to a maximum of one, providing a relative abundance. To mitigate over-assignment to sorted TME cell types, we included unsorted cell types (neoplastic + oligodendrocyte precursor cell with high transcriptomic similarity) in this deconvolution step as a ‘block’ effect. Joint feature plots (tumour and TME) were generated using a custom modification of the SpatialFeaturePlotBlend() source function. The original repository is available at https://github.com/george-hall-ucl/SpatialFeaturePlotBlend. For the TME population-level quantification, for each spot, the weights of all deconvolved populations were normalized to sum to one, thereby representing the relative contribution of each cell population within that specific spot. Subsequently, these normalized weights were plotted across the three metabolic states.

### MSI and spatial transcriptomic data co-registration

Total ion count-normalized MSI data were extracted using the SCiLS Lab API (v.2022b; Bruker Daltonik), and metabolic labels were spatially smoothed to provide coherent spatial regions. Immediately neighbouring pixels (≤8) were identified for each MSI pixel, and across five iterations, for each pixel that had at least two neighbours and for which the majority of neighbours were assigned a different label, the label was replaced with the majority label. An image was created using spatial coordinates, in which pixels were coloured using the first three principal components as RGB channels. Landmarks were identified between this MSI dimensionality reduction image and the H&E image of the corresponding tissue section, and subsequently between the H&E image and the corresponding H&E image of the contiguous tissue section used for spatial transcriptomic analysis. These landmarks were then used to map the MSI coordinates onto the spatial transcriptomic coordinate space using an affine transform. MSI labels were finally transferred to spots on the spatial transcriptomic image using the *k*-nearest neighbours algorithm (*k* = 3).

### Defining metabolic regions on MSI

Regions were defined using *k*-means clustering and fitted using the *Kmeans* function in the *amap* R package and performed independently on the neurosphere, human and metastases datasets. The values for each of the seven ^13^C-labelled metabolites used for metabolic clustering were standardised into *z*-scores (by subtracting the metabolite’s mean and dividing by the metabolite’s standard deviation). Each pixel was then assigned to three or four groups, using *k*-means clustering with Manhattan distances.

### IMC and immunohistochemistry

Antibodies used for immunohistochemistry are described in Supplementary Table 5. Antibodies were tagged using the Fluidigm Maxpar Antibody Labelling Kit. Slides were fixed with 4% paraformaldehyde in PBS for 10 min, washed three times in PBS, permeabilized using a 1:1,000 dilution of Triton X-100 in casein solution, washed another three times in PBS and then blocked for 30 min with casein solution (Thermo Fisher). Antibodies were diluted in casein solution, and the slides were incubated overnight at 4 °C. The slide was then washed three times in PBS, and nuclei were stained with DNA intercalator-iridium (Fluidigm) at a dilution of 1:400 in PBS for 30 min. The slide was washed three times in PBS, 30 s in deionized water and then dried at room temperature. A region for IMC analysis was selected using consecutive H&E-stained sections and the DESI-MSI data. IMC analysis was performed using a Hyperion Instrument (Fluidigm Corporation) with an ablation energy of 4 db and an ablation frequency of 200 Hz. IMC images were produced using MCD viewer (v.1.0; Fluidigm), and analysis was performed using HALO (Indica Labs).

Tumour-bearing rat brains were snap-frozen in liquid nitrogen and sectioned at 6 μm thickness for immunohistochemistry analysis using Leica’s Polymer Refine Kit (antibodies listed in Supplementary Table 6). Images were analysed using Aperio image-viewing software and HALO (v.3.6.4134.137).

### HALO image analysis

HALO (v.3.6.4134.137) and HighPlex FL (v.4.1.3) modules were used for automated image analysis. Optical densities for weakly, moderately and strongly stained cells used for the automated quantitative analysis of scanned sections were as follows: Ki67 – (nuclear) 7, 40.7522, 54.385, p53 – (nuclear) 1.8, 3.5929, 5.1327, CC3 – (cytoplasm) 1.9427, 4.646, 6.1947, Vimentin – (cytoplasm) 13.3343, 25.7522, 41.2035, CD3 – (nuclear) 2.2832, 32.6875, 63, GZMB – (nuclear) 0.6956, 0.8673, 1.6106, CD4 – (cytoplasm) 3.9823, 6.7699, 10.354, CD8A – (nuclear) 1.6327, 18.7679, 26, CD68 – (cytoplasm) 10.4106, 45.9027, 78.6903, CD45 – (cytoplasm) 2, 5.3363, 7.115, ASMA – (cytoplasm) 2.8, 12.1327, 18.6239, CD31 – (cytoplasm) 2.274, 4.5841, 6.876, panCK – (cytoplasm) 1.836, 2.8673, 3.9823, Collagen I – (cytoplasm) 28.293, 104.7301, 179.58.

Five cellular phenotypes were identified: CD4^+^ helper T cells (CD45^+^CD4^+^CD3^+^); cytotoxic T cells (CD3^+^CD45^+^CD8A^+^); activated cytotoxic T cells (CD3^+^CD45^+^CD8A^+^GZMB^+^); natural killer cells and neutrophils (CD45^+^GZMB^+^); and macrophages and microglia (CD68^+^). A random forest classifier was used to distinguish vessels and non-vessels within the images. Large vessels were positive for Collagen I and CD31. Annotations were created manually on several images, and these were used to train the classifier. The five cellular phenotypes were plotted spatially, three 60 µm bands travelling out from the vessels were defined and the total number of cells displaying these cellular phenotypes in each band was determined.

### Statistical analyses

Sample size for human tumour collection was determined intra-operatively based on patient and tumour factors (for example, proximity to eloquent brain). A minimum of six samples were collected for each tumour region. For xenograft and neurosphere studies, a minimum of three independent biological replicates were used (three different rats; three different cell line passages with a minimum of six technical replicates). No randomization or blinding was used, and all data were included in the analysis. Statistical analyses were performed using GraphPad Prism (v.10.0.3), R (v.4.3.0) and SCiLS. Bioinformatics analyses were performed in R. Statistical tests were two-sided unless stated otherwise. Student’s *t*-tests and one-way ANOVA with Tukey’s multiplicity correction were used to test the equality of means between two or more groups, respectively.

### Reporting summary

Further information on research design is available in the Nature Portfolio Reporting Summary linked to this article.

## Supplementary information

Supplementary Information Supplementary Tables 1–6.

Reporting Summary

## Source data

Source Data Fig. 1 Statistical source data.

Source Data Fig. 2 Statistical source data.

Source Data Fig. 3 Statistical source data.

Source Data Fig. 4 Statistical source data.

Source Data Extended Data Fig. 1 Statistical source data.

Source Data Extended Data Fig. 2 Statistical source data.

Source Data Extended Data Fig. 3 Statistical source data.

Source Data Extended Data Fig. 4 Statistical source data.

Source Data Extended Data Fig. 5 Statistical source data.

Source Data Extended Data Fig. 5 HALO IMC analysis output.

Source Data Extended Data Fig. 7 Statistical source data.

Source Data Extended Data Fig. 8 Statistical source data.

## Data Availability

The raw and processed RNA sequencing and MSI data that are not derived from GB patient material are available at the following links: RNA sequencing data are available at https://www.ncbi.nlm.nih.gov/geo/GSE288836; the spatial transcriptomics RNA object (seuraT_obj.RDATA) is available at 10.17863/CAM.116214; and the MSI data are available at https://www.ebi.ac.uk/metabolights/MTBLS12176. The authors defer deposition of raw MSI data derived from GB patient material to ensure compliance with legal requirements of the University of Cambridge and Cambridge University Hospitals NHS Foundation Trust and avoid compromising the privacy of the study participants. Requests for raw data can be referred to the corresponding authors; these will be reviewed within ten working days in consultation with the institutional R&D, which will determine the terms of a data transfer agreement between the recipient institution, the University of Cambridge and Cambridge University Hospitals NHS Foundation Trust. Source data are provided with this paper.
