# Supplementary material for: Cell-intrinsic metabolic phenotypes identified in patients with glioblastoma, using mass spectrometry imaging of 13C-labelled glucose metabolism
Source: Nat Metab. 2025 May 19;7(5):928–39. doi: 10.1038/s42255-025-01293-y (PMC12116388; doi:10.1038/s42255-025-01293-y)
Supplement: Supplementary file 1 — Supplementary Tables 1–6. [file 42255_2025_1293_MOESM1_ESM.pdf]

nature metabolism

Supplementary information https://doi.org/10.1038/s42255-025-01293-y

Cell-intrinsic metabolic phenotypes identified in patients with glioblastoma, using mass spectrometry imaging of ^{13}C-labelled glucose metabolism

In the format provided by the authors and unedited

Cancer Research UK Rosetta Grand Challenge consortium

- Owen J. Sansom[1,2]
- Josephine Bunch[4]
- Andrew Campbell[1]
- Arafath Najumudeen[1]
- Gregory Hamm[3]
- Alan M. Race[3]
- Ian Gilmore[4]
- Greg McMahon[4]
- Paul Grant[4]
- Bin Yan[4]
- Rory T. Steven[4]
- Adam J. Taylor[4]
- Chelsea J. Nikula[4]
- Efstathios Elia[4]
- Spencer Thomas[4]
- Catherine Munteanu[4]
- Ala Al-Afeef[4]
- Alex Dexter[4]
- Teresa Murta[4]
- Amy Burton[4]
- Jean-Luc Vorng[4]
- Xavier Loizeau[4]
- Weiwei Zhou[4]
- Ammar Nasif[4]
- Ariadna Gonzalez[4]
- Hanifa Koquna[4,6]
- Martin Metodiev[4]
- Melina Kyriazi[4]
- Junting Zhang[4]
- Lucas Zeiger[1]
- Johan Vande-Voorde[1]
- Jennifer Morton[1]
- David Sumpton[1]
- David Y. Lewis[1]
- Dmitry Soloviev[1]
- Zoltan Takats[6]
- Nicole Strimatter
- Vincen Wu[6]
- Yuchen Xiang[6]
- Daniel McGill[6]
- Stefania Maneta-Stravrakaki[6]
- Jaynisha Mistry[6]
- Emine Kazanc[6]
- Mariia Yuneva[8]
- Yulia Panina[8]
- Chandan Seth Nanda[8]
- Peter Kreuzaler[8]
- Avinash Ghanate[8]

- Richard J. A. Goodwin[3,7]
- Simon T. Barry[5]
- Stephanie Ling[5]
- Jack Richings[5]
- Kevin Brindle[9]
- Anastasia Tsyben[9]
- George Poulogiannis[10]
- Amit Gupta[10]
- Aurelien Tripp[10]
- Evi Karali[10]
- Nikolaos Koundouros[10]
- Thanasis Tsalikis[10]
- John Marshall[11]
- Magali Garrett[12]
- Harry Hall[12]
- Hanifa Koguna[4,6]
- Najah Islam[6]
- Erica Montezuma[6]
- Amy Spicer-Hadlington[8]
- James McKenzie[6]
- Alvaro Perdones Monteiro[6]
- Manas Kohli[10]
- Ala Amgheib[6]
- Caroline Sproat[11]
- Daria Thompson[8]
- Dipa Gurung[6]
- Emma White[6]
- Paolo Inglese[6]
- Renata Filipe Soares[13]
- Shreya Sharma[11]
- Avinash Ghhanate[8]
- Adele Savage[6]
- Ali Demircali[6]
- Pamela Pruski[6]
- Jinshi Zao[6]
- Petra Paiz[6]
- Jyotsna Rao[9]
- Liam Poynter[6]
- Maria Fala[9]
- Alan Wright[9]
- Nicole Strittmatter[13]

[1]Cancer Research UK Beatson Institute, Glasgow, UK. [2]Institute of Cancer Sciences, University of Glasgow, Glasgow, UK. [3]Imaging and data Analytics, Clinical Pharmacology and Safety Sciences, R&D, AstraZeneca, Cambridge, UK. [4]National Physical Laboratory, Teddington, Middlesex, UK. [5]Bioscience, Oncology R&D, AstraZeneca, Cambridge, UK. [6]Department of Metabolism, Imperial College London, London, UK. [7]Institute of Infection, Immunity and Inflammation, College of Medical, Veterinary and Life Sciences, University of Glasgow, Glasgow, UK. [8]The Francis Crick Institute, London, UK. [9]University of

Cambridge, Cambridge, UK. [10]*Institute of Cancer Research, London, UK.* [11]*Barts Cancer Institute, London, UK.* [12]*Patient advocates, London, UK.* [13]*School of Life Sciences, University of Dundee, Dundee, UK.* [13]*TUM School of Natural Sciences, Technical University of Munich, Germany.*

Supplementary Data Tables

	Metabolite	Adduct	Theoretical *m/z*	Measured *m/z*	Error [ppm]
DESI					
	[U-^{12}C]glucose	[M-H]-	179.056114	179.056	-0.636671921
	[U-^{12}C]glucose	[M+Cl]-	215.032792	215.03282	0.130212698
	[U-^{12}C]lactate	[M-H]-	89.024419	89.0244	-0.213424589
	[U-^{12}C]glutamate	[M-H]-	146.045883	146.0458	-0.568314548
	[U-^{13}C]glucose	[M-H]-	185.076244	185.0762	-0.237739858
	[U-^{13}C]glucose	[M+Cl]-	221.052922	221.05285	-0.325713858
	[U-^{13}C]pyruvate	[M-H]-	90.018834	90.0188	-0.377698738
	[U-^{13}C]lactate	[M-H]-	92.034484	92.0344	-0.912701374
	[$^{13}C_2$]fumarate	[M-H]-	117.010394	117.0104	0.051277496
	[$^{13}C_2$]succinate	[M-H]-	119.026044	119.026	-0.369666995
	[$^{13}C_2$]malate	[M-H]-	135.020959	135.021	0.30365656
	[$^{13}C_2$]glutamate	[M-H]-	148.052593	148.0526	0.047280496
	[$^{13}C_2$]glutamine	[M-H]-	147.068577	147.0685	-0.523565275
	ascorbic acid	[M-H]-	175.024814	175.0247	-0.651336216
	Dehydroascorbic	[M-H]-	173.009164	173.009	-0.947926666
MALDI					
	GSH	[M-H]-	306.076533	306.072	-14.81002139
	GSSG	[M-H]-	611.144692	611.148	5.412793473
	PCr	[M-H]-	210.028534	210.025	-16.82628514
	ADP	[M-H]-	426.022145	426.014	-19.11872445
	ATP	[M-H]-	505.988478	505.985	-6.873674305

Table S1. Mass errors on metabolite ions in the human GB dataset.

		GB1																	
**Metabolite**	**Isotope**	**Region 1**	**Region 1**	**Region 1**	**Region 1**	**Region 1**	**Region 1**	**Region 2**	**Region 2**	**Region 2**	**Region 2**	**Region 2**	**Region 2**	**Region 2**	**Region 3**	**Region 3**	**Region 3**	**Region 3**	**Region 3**
**Pyruvate**	**m+0**	78.9991837	70.2366638	24.4254532	44.2851563	27.1803246	23.7652378	50.7086258	82.8216858	79.1899796	28.3437767	45.2390175	24.2426662	24.8160553	50.1244621	21.8434658	21.1654205	18.9320755	20.5852642
	**m+1**	1.48086226	1.81099248	0.24412802	0.3928169	0.25406593	0.19604923	1.07805681	1.7534411	2.18101001	0.42975432	0.52448815	0.19835426	0.191548	0.99431479	0.3144232	0.22591317	0.11957863	0.05944376
	**m+2**	0.82742542	1.75026143	1.31504345	0.45975634	0.8493734	1.41227686	1.92554605	0.84719884	2.31087041	1.4369638	0.68341058	1.01698244	1.0917083	2.50976396	1.47734141	1.75434685	1.26810431	1.30198109
**Lactate**	**m+0**	1417.99597	1535.00391	1483.61597	1240.72595	1333.47644	1509.42834	1644.38135	1439.42175	1676.53394	1489.71252	1311.90454	1314.87817	1381.18262	1771.92249	1525.30615	1597.37805	1512.37939	1430.10437
**Pyruvate**	**m+3**	0.54508185	1.97689033	1.20639682	1.55636811	0.83358526	0.60223836	2.21407199	0.61910105	2.50443316	1.68368995	1.99464309	1.01543558	0.86798668	2.41808629	1.62150776	2.16186619	0.9807207	1.12168264
**Lactate**	**m+1**	65.2783813	91.7389832	89.6184082	66.9832077	72.0751495	72.6371994	100.644386	69.6409073	104.173035	92.0000229	73.1794281	74.3204956	73.2866592	116.953873	94.8180313	82.3907318	81.4857712	69.7113571
	**m+2**	19.8812847	41.0646133	47.947937	30.6761513	34.8694038	26.2637177	51.6288681	22.5606861	49.4295998	50.5146599	32.9125862	38.9436913	34.18647	63.0151329	53.3891487	45.3837662	40.6595497	32.1952705
	**m+3**	96.2826233	212.188004	366.579071	236.147324	294.057434	208.738647	321.301086	104.534363	246.54747	386.139679	225.347076	319.145447	312.059906	390.866913	420.158386	509.709717	395.483307	403.233337
**Fumarate**	**m+0**	94.6044235	39.1883278	60.8476563	51.5257645	45.2821159	39.0515938	77.1000595	132.928757	52.7264214	86.2986145	65.5744324	51.7566071	45.9978752	82.7060928	60.5141792	15.0288315	39.4351845	29.9574184
	**m+1**	3.84941316	1.24201584	3.3832128	1.27634537	1.66942155	1.98033321	3.33417916	6.21372509	2.1358304	5.19422817	2.58380151	2.55873013	2.33188987	3.48897266	3.63714838	0.22209452	1.42818749	1.24863541
	**m+2**	0.61680371	0.08854344	1.38676345	0.12495752	0.31745258	0.67172474	0.59410924	1.7096647	0.28808412	2.00632286	0.27254963	0.61375779	0.83687443	1.04691994	1.37989163	0.02709664	0.37536183	0.30727884
**Succinate**	**m+0**	236.811539	65.9852829	126.250954	66.2914734	64.3253784	64.6240158	115.310059	341.327576	93.0605316	163.891937	86.8933868	73.6068344	72.3919907	120.868721	132.968445	22.308754	59.2064781	47.1994896
**Fumarate**	**m+3**	0.08578623	0.01174058	0.14453587	0.02804987	0.02149862	0.0405264	0.15142028	0.2272194	0.04057592	0.18312426	0.04475911	0.02769053	0.05240374	0.2748476	0.19347696	0.01465625	0	0.04110514
**Succinate**	**m+1**	11.1789417	2.5678215	8.99479008	2.3475368	3.4980607	4.70161104	5.51960039	16.3059731	4.19002676	11.5665636	4.02799845	4.63787127	5.33738565	5.45655727	9.52698612	0.36413664	3.76024461	3.50009537
	**m+2**	4.1259079	0.66240722	8.1404686	0.69424468	1.9064759	3.74468398	2.55313945	7.15364265	1.29595399	9.84502411	1.66491687	2.90197039	4.40427828	2.36616898	8.34218502	0.10962459	2.59196162	2.96801496
	**m+3**	0.00655785	0.00071052	0.17042755	0.00165876	0.01505597	0.04074277	0.03208087	0.02549321	0	0.17782865	0	0.0241548	0.07027072	0	0.14686294	0	0	0.03066035
**Malate**	**m+0**	255.953796	65.590332	155.496567	52.1345558	68.512352	69.4706497	95.3606644	421.28299	103.366257	247.503677	82.7674866	89.4708939	86.7473526	98.7133789	177.843674	11.6182528	73.2934418	49.5077362
	**m+1**	0.16933957	0.00294658	0.00204295	0	0.00879015	0.00152829	0.01809332	0.19984113	0.01351749	0.00325992	0	0	0	0	0.00186613	0	0	0
	**m+2**	6.323627	1.13567531	7.75401354	1.34028554	3.44620657	4.49585676	1.97176325	11.2113447	2.5816443	11.8480759	2.72482848	5.44022274	6.30293465	2.67478323	8.64628983	0.12472567	4.06334496	3.99363112
	**m+3**	3.28159499	0.68092132	2.40543795	0.3667807	0.52142781	0.78444141	1.35310042	6.54098558	1.34391677	3.901474	0.92824042	0.69938976	1.33863509	1.21589661	2.85146451	0.0995461	0.45451343	0.73423493
**Glucose**	**m+0**	745.376648	515.092468	125.550232	360.778656	658.573486	313.404907	599.587463	736.88324	517.59967	177.833191	453.961121	622.26593	339.960144	483.506287	101.698792	229.991974	398.971436	183.709702
	**m+1**	50.6832809	34.5966492	8.26246929	24.9605618	45.8370819	21.2978268	42.4822388	50.010582	34.5131836	11.9448996	31.9567051	43.9721107	23.3022785	33.4961662	6.79819345	15.980484	28.4818192	13.0264444
	**m+2**	4.69980288	4.18506098	0.32600093	2.11911941	4.74699306	2.02972317	5.09376574	4.73229885	4.4019866	0.63454002	3.41968346	4.67072153	2.59514403	3.34382749	0.24572501	1.60814166	2.88172317	0.98698479
	**m+3**	8.66395187	7.23439217	1.07420421	6.30425167	10.5574026	5.42169905	10.048811	8.64987373	7.24979019	2.0933814	8.42600822	10.1320086	6.51910448	7.88647127	0.86578238	4.14777565	6.39723682	2.68801594
	**m+5**	12.0400658	9.52741432	1.77102697	9.2177515	13.7255592	6.91570377	17.3056316	11.7575321	9.20721054	3.07735038	11.9743385	12.8060741	8.20265102	13.7229891	1.41647077	5.88362312	8.28083324	3.8261838
	**m+6**	170.380005	135.039017	32.3291321	128.402847	199.095657	103.833252	199.460587	166.536621	132.330627	48.1238289	158.018677	191.059372	122.631653	149.841492	25.5463276	88.0132599	121.76535	60.2649002
**Glutamate**	**m+0**	273.682983	195.838608	160.764618	182.249252	178.297394	110.562531	302.327545	393.799377	265.450836	232.86821	247.619995	233.435471	182.742294	302.46167	166.211517	75.74086	167.236511	138.944901
	**m+1**	16.2809486	12.0041723	11.8622446	13.2104149	13.7294693	9.01951122	17.9133072	24.0086498	16.6344643	17.1113167	18.4384441	18.8330135	15.587184	18.2099247	12.4762459	4.8605237	13.3994207	12.7193413
	**m+2**	8.70333385	6.56019545	11.7050123	12.202632	14.0452576	9.99817753	13.4740238	13.4237528	10.1813517	16.8462563	16.9187164	19.262991	18.3028393	13.6864243	12.2887192	4.93153954	13.3995867	15.7917347
**Glutamine**	**m+0**	291.244659	345.724121	163.015106	306.647461	194.463516	138.60907	298.216156	382.503845	483.045227	224.5	412.6008	217.399612	154.208115	307.685669	168.495239	82.3930206	157.251984	91.6339188
	**m+1**	15.3670521	18.6464977	9.22170639	17.349678	12.8982496	9.65744209	15.6242933	20.1249027	25.951746	12.9553003	23.9396019	14.8911562	11.4869528	15.9307404	9.88836479	4.18700695	10.6309061	6.91298866
	**m+2**	0.72649801	1.65116978	3.084939	4.67243958	7.48218012	6.63647699	2.37374806	1.28785408	3.0085113	4.53566122	7.22725582	9.09069443	9.17063904	2.45469546	3.38427448	0.95398545	6.00199032	6.34018946

GB2																	
**Region 1**	**Region 1**	**Region 1**	**Region 1**	**Region 1**	**Region 1**	**Region 2**	**Region 2**	**Region 2**	**Region 2**	**Region 2**	**Region 3**	**Region 3**	**Region 3**	**Region 3**	**Region 3**	**Region 3**	**Region 3**
13.3787575	25.7180729	10.6838799	17.7564411	11.5358906	26.6744652	19.9553585	29.0239563	13.763258	21.6369553	30.8299656	14.0664949	24.0424232	14.9206657	20.9490128	14.2204571	22.0877342	26.2893085
0.06874543	0.43545312	0	0.15654713	0.09440062	0.31305021	0.05879882	0.54268736	0.25791937	0.26556459	0.49732026	0.07082883	0.43391135	0.23039541	0.16436021	0.20409921	0.3421039	0.4038853
0.32047188	1.06492198	0.78401273	0.54042798	0.20810579	0.31666183	0.23526354	1.71745026	2.48636341	0.74440861	1.69125092	0.73672801	1.85959315	2.16684961	0.76258916	0.98572826	0.87206084	1.9279058
728.188293	924.622803	1268.52319	686.63739	484.345093	668.287903	871.171753	1336.83484	1762.03796	779.786255	1515.41772	989.803101	1403.42896	1682.82251	829.776123	899.69342	895.655701	1615.35193
0.96229482	2.72785354	1.67798162	1.561638	0.63837874	2.71786141	1.23892343	3.20605564	2.08658409	1.85344172	3.50956583	1.42061484	2.95536423	2.67169452	2.09291005	2.17972088	3.52197123	2.60911202
38.049633	48.1173782	68.1418915	44.9404793	28.5644817	36.4664383	47.2998123	70.3818359	83.5015793	54.4658203	88.0986481	55.6284714	73.7324066	85.4104309	57.2271042	54.6507721	52.4241104	89.8454056
25.3385067	28.2224483	49.6778107	33.7140884	22.6418114	25.8869267	29.1517925	46.1478462	45.5614471	42.0990562	60.6241226	39.1414452	46.438427	52.910038	44.8972626	45.8052483	38.2840462	56.0022621
300.299469	322.337799	587.152344	304.771393	299.274536	333.563782	329.104095	546.732483	644.700806	362.159302	567.875305	414.721436	557.058838	705.935547	401.080963	592.550964	490.997101	522.147339
10.6854744	6.14914799	13.2455235	22.8992958	3.7895689	6.06887341	14.1895189	18.7381668	18.9729214	29.3177395	37.9944458	16.2372131	13.8257723	14.8504753	29.2164783	5.90070772	9.49074268	24.9296761
0.13497159	0.04436611	0	0.81310326	0.03027512	0.03913793	0.29313198	0.96763003	1.08775198	1.2960391	1.84269047	0.47155926	0.37571749	0.7232011	1.10503721	0.06743767	0.13123213	0.75372905
0.06419798	0.00987797	0.34317374	0.34655532	0.00617964	0.00424995	0.36666921	0.5203855	0.82825428	0.50674498	0.94203895	0.30690405	0.15715739	0.57135546	0.71547353	0.01826699	0.01336464	0.23603676
16.0012207	10.1386042	36.2127609	32.0486984	6.78093386	9.95476055	26.4132538	30.6529179	52.3966408	40.5415649	66.7960587	23.8861713	23.7294559	38.5363235	42.2511063	10.0967007	13.8759851	42.7850647
0.01483563	0	0.36586273	0.04495332	0	0.00719306	0	0.13088864	0.10394597	0.04121178	0.26512399	0.0739031	0.02933656	0.07728791	0.04919882	0.00689819	0.00263384	0.05439685
0.35017824	0.16243368	2.91837645	1.42104721	0.08937862	0.11368086	1.0317961	1.63891709	4.23961878	1.851282	3.82405448	0.90727967	1.00355697	2.90231347	1.90688837	0.3261748	0.44301456	1.75888789
0.26902467	0.07291275	3.43167019	0.6644991	0.05517084	0.06199558	0.65862346	1.53212917	5.07967043	1.05477059	3.33988261	1.14131773	0.87005579	3.66477966	1.3138057	0.28272173	0.3488085	0.87379277
0.00709994	0	1.07941806	0.00838803	0.00067658	0	0	0.02475418	1.16508675	0.01008594	0.23210417	0	0.03329561	0.76394445	0.01525815	0.01121826	0.00448061	0.04335847
9.94722843	6.10888147	24.3250389	23.2532864	3.45606732	3.46410775	23.8932056	42.9394569	46.9463692	30.0366936	124.578697	23.1004219	22.9088116	34.5104446	29.4646664	6.29461527	6.54638863	61.4888992
0	0	0	0	0	0.00155952	0	0	0	0	0	0	0.00120989	0	0	0	0	0
0.46121183	0.08434454	3.03475261	1.49009275	0.08026624	0.06581756	1.07935059	2.91803432	4.95412207	2.19851041	9.47500992	1.64942515	1.33388209	3.56650019	2.3186667	0.32856768	0.33262062	3.12880492
0.11034065	0.02787254	1.28790271	0.35238597	0.03667284	0.02606912	0.24899341	2.03029156	1.56129575	0.47003442	4.99014711	0.54284686	0.68364507	1.28116	0.38896978	0.13486765	0.11457907	1.54030681
84.5499268	169.21579	53.2944412	205.929321	41.5406113	139.544769	102.710419	158.976883	57.7422066	231.690933	224.669601	94.8400803	150.67485	54.9097252	216.674591	74.7997818	168.628006	198.409027
5.78758669	11.5248365	3.06623626	14.6294584	2.27374053	9.8428545	5.76971626	10.1034746	3.69606924	16.1416016	15.0832777	6.80755854	9.91550827	3.48269176	14.9242077	4.78917933	11.7978973	13.6641054
1.4578172	2.27068162	0.38029	3.7243042	0.25851831	1.91413093	1.24605286	1.51918936	0.08283433	4.2396183	2.29746532	1.49907064	1.57197297	0.2084401	3.77141929	0.96649039	2.91660905	2.50445223
2.23434377	3.93455815	0	6.72962952	0.55975759	4.12323427	2.07833862	2.3161695	0.14318818	7.60060501	4.6859498	2.39512229	2.71987414	0.33456993	7.07078505	1.59862328	5.48976564	4.73178005
0.83109677	2.78179646	0	9.26752281	0.55753088	6.21773815	3.00784421	1.79153454	0.34013435	13.5815315	10.0232573	0.46288389	1.46892202	0.53496855	11.1076269	0.64603323	3.98535466	9.76245785
70.9867859	124.630432	8.64682293	175.292969	26.156889	128.537704	80.2535553	78.5339661	8.81589508	187.407333	118.537376	77.0896759	85.8117981	14.1992426	175.90863	47.4591484	153.077332	122.131714
39.074646	43.9466515	139.974136	73.4415436	23.7337894	38.0357437	87.2393036	126.668755	142.847733	106.53231	216.954193	63.313942	100.319221	108.956902	104.603035	42.2163734	54.1847115	121.545349
2.56001019	3.19862223	9.84561729	6.44764948	1.72481906	3.31586671	5.08356524	9.73208714	11.8068314	9.44886208	16.8497391	4.67625666	7.39492083	8.51404667	9.57598019	3.8590703	5.57746124	8.2601223
4.44694805	4.23015833	17.5553474	10.3742199	3.61379385	5.53510666	11.6020193	13.7418365	18.8304005	15.7268524	24.9114723	8.37348843	9.40491009	13.9005299	15.4193583	6.68463373	8.75246143	9.54763222
48.8571548	91.3419342	145.02626	110.387909	47.2351112	71.4218521	86.4705505	195.158493	98.2239532	134.934586	540.516541	70.086998	150.296204	101.612953	136.911636	80.9197388	100.058449	396.283569
2.86200476	5.27480412	10.3414516	8.52053261	2.72035241	4.46367836	5.03509855	12.6513643	7.45997143	10.6986256	35.0183144	4.20577765	9.179142	7.77473927	10.6080809	5.32417917	6.91151571	23.9797039
2.44739056	3.14153171	13.3521004	9.94195557	2.72865868	3.87456584	5.12821293	10.8334436	10.023695	13.0612307	27.9700699	4.80399179	6.46082354	10.6402521	12.6269999	5.05792665	6.10092831	14.2290955

Table S2. Mass isotopologue distributions of all detectable labelled metabolites in GB1 and GB2 tumour samples.

Tumour	Time (min)	Glutamine	[$^{13}C_2$]glutamine	Serine	Lactate	[U-^{13}C]lactate	Pyruvate	[U-^{13}C]pyruvate	Malate	[$^{13}C_2$]malate	Glutamic acid	[$^{13}C_2$]glutamate	Glucose	[U-^{13}C]glucose
**GB1**	0	8,488,974	35,766	41,374	2,417,571	975	108,376	ND	107,366	ND	106,348	9,822	5040187.49	448897.2433
**GB1**	30	8,678,183	55,176	36,207	4,576,187	76,191	210,019	1,986	189,831	3,576	136,443	ND	4037706.07	1615183.563
**GB1**	60	8,885,515	86,700	35,615	2,878,676	111,242	158,471	4,640	174,209	3,219	141,328	29,102	4319893.55	2006283.003
**GB1**	90	8,991,838	138,866	35,388	1,864,968	119,912	127,773	6,604	110,416	6,164	90,621	ND	3939648.97	2355203.397
**GB1**	120	8,855,463	164,829	37,508	3,789,696	273,599	254,460	15,559	208,384	10,925	178,849	ND	3781662.05	2330591.923
**GB1**	130	8,903,307	185,703	36,311	4,714,241	340,972	339,106	24,326	308,580	7,764	208,112	ND	3691290.05	2180081.907
**GB2**	0	9,994,363	45,665	55,650	5,333,179	ND	120,342	ND	110,826	4,450	155,747	ND	3585620.88	#DIV/0!
**GB2**	30	9,646,378	58,016	52,570	3,988,172	123,009	160,324	3,331	123,746	ND	93,846	43,856	3182060.59	2421385.723
**GB2**	65	8,659,828	100,322	63,589	5,463,861	309,137	279,812	14,652	120,293	ND	93,461	4,962	2500519.81	2536754.903
**GB2**	90	8,869,511	161,009	42,956	5,031,684	490,620	274,854	24,258	121,135	3,299	131,321	11,939	2491925.99	2882720.207
**GB2**	120	7,819,478	230,332	36,324	2,644,420	419,053	169,577	24,990	70,821	ND	109,868	20,994	2281782.07	3193371.013
**GB2**	130	7,627,721	262,727	36,826	2,528,348	482,060	212,729	34,274	98,683	2,172	132,450	13,439	2128510.01	2605086.853
**Metastasis**	0	11,655,684	80,976	44,073	3,297,545	137,074	146,390	3,749	337,511	3,395	148,021	ND	3741229.42	1753079.027
**Metastasis**	30	11,713,998	185,317	39,734	3,902,706	286,885	233,535	14,487	285,940	ND	147,919	22,016	3263028.94	2047254.917
**Metastasis**	60	10,614,064	253,151	36,583	2,605,872	238,764	153,207	10,330	164,392	3,153	88,124	57,569	3054498.07	2276855.087
**Metastasis**	90	10,117,090	66,822	37,451	2,515,020	110,973	128,563	3,167	230,633	4,517	89,834	9,189	3598955.51	1695703.117
**Metastasis**	120	10,203,660	339,243	32,531	4,243,029	494,633	298,405	32,751	256,285	5,816	203,303	51,561	2802720.72	2506409.843
**Metastasis**	130	10,144,353	417,133	34,262	4,289,442	631,262	302,783	41,729	247,739	5,459	180,168	76,234	2639408.83	2522768.833

Table S3. Relative concentrations of labelled and unlabelled metabolites measured in patients’ plasma using LC-MS.

a

ID	setSize	NES
E2F_TARGETS	200	2.602632
MYC_TARGETS_V1	200	2.474932
MYC_TARGETS_V2	58	2.528370
HEDGEHOG_SIGNALING	35	-2.240655
G2M_CHECKPOINT	199	1.850801
HYPOXIA	191	1.678919
GLYCOLYSIS	190	1.729728
INTERFERON_ALPHA_RESPONSE	97	1.794424
INFLAMMATORY_RESPONSE	165	1.679769
MTORC1_SIGNALING	198	1.583894

b

ID	setSize	NES
MYC_TARGETS_V1	200	-3.798247
E2F_TARGETS	200	-3.579663
OXIDATIVE_PHOSPHORYLATION	200	-3.139455
EPITHELIAL_MESENCHYMAL_TRANSITION	196	2.874561
G2M_CHECKPOINT	199	-2.963346
UV_RESPONSE_DN	144	2.476548
MYC_TARGETS_V2	58	-2.894760
DNA_REPAIR	149	-2.510455
MTORC1_SIGNALING	198	-2.131432
COAGULATION	105	2.268616

Table S4. GSEA of the PC1 loadings for neurospheres grown under normoxia (a) and hypoxia (b).

Target	Label	Clone	Dilution	Product Code	Manufacturer
αSMA	141Pr	1A4	1:50	3141017D	Standard Biotools
Vimentin	143Nd	RV202	1:100	3143029D	Standard Biotools
CD31	145Nd	JC/70A	1:50	ab264090	Abcam
Cleaved Caspase 3	147Sm	E83-77	1:50	ab208003	Abcam
Pan-CK	148Nd	C11	1:50	3148020D	Standard Biotools
CD45	152Sm	D9M8I	1:100	3152018D	Standard Biotools
CD3	154Sm	UCHT1	1:50	3170022D	Standard Biotools
CD4	156Gd	RPA-T4	1:50	3156036D	Standard Biotools
CD68	159Tb	KP1	1:100	3159035D	Standard Biotools
CD8	162Dy	RPA-T8	1:50	3162036D	Standard Biotools
Ki67	168 Er	B56	1:50	3168022D	Standard Biotools
Collagen I	169Tm	Polyclonal	1:100	3169023D	Standard Biotools
Granzyme B	167 ER	GB11	1:50	3167023D	Standard Biotools
p53	165	DO-7	1:50	MA5-12557	Thermo Fisher

Table S5. IMC antibodies and product information.

Target	Species	Dilution/Concentration	Product Code	Manufacturer
Ki67	Human	0.046 μg/ml	M7240	Agilent Dako
CC3	Human	1:250	9664	Cell Signaling Technology
CD31	Rat	1.128 μg/ml	ab182981	Abcam

Table S6. IHC antibodies and product information.
