# Supplementary material for: Cell-intrinsic metabolic phenotypes identified in patients with glioblastoma, using mass spectrometry imaging of 13C-labelled glucose metabolism
Source: Nat Metab. 2025 May 19;7(5):928–39. doi: 10.1038/s42255-025-01293-y (PMC12116388; doi:10.1038/s42255-025-01293-y)
Supplement: Supplementary file 2 — Reporting Summary [file 42255_2025_1293_MOESM2_ESM.pdf]

nature portfolio

Corresponding author(s): Kevin Brindle, Richard Mair

Last updated by author(s): Jan 10, 2025

Reporting Summary

Nature Portfolio wishes to improve the reproducibility of the work that we publish. This form provides structure for consistency and transparency in reporting. For further information on Nature Portfolio policies, see our Editorial Policies and the Editorial Policy Checklist.

Statistics

For all statistical analyses, confirm that the following items are present in the figure legend, table legend, main text, or Methods section.

n/a	Confirmed	
☐	☒	The exact sample size (*n*) for each experimental group/condition, given as a discrete number and unit of measurement
☐	☒	A statement on whether measurements were taken from distinct samples or whether the same sample was measured repeatedly
☐	☒	The statistical test(s) used AND whether they are one- or two-sided<br>*Only common tests should be described solely by name; describe more complex techniques in the Methods section.*
☐	☒	A description of all covariates tested
☐	☒	A description of any assumptions or corrections, such as tests of normality and adjustment for multiple comparisons
☐	☒	A full description of the statistical parameters including central tendency (e.g. means) or other basic estimates (e.g. regression coefficient) AND variation (e.g. standard deviation) or associated estimates of uncertainty (e.g. confidence intervals)
☐	☒	For null hypothesis testing, the test statistic (e.g. *F*, *t*, *r*) with confidence intervals, effect sizes, degrees of freedom and *P* value noted<br>*Give P values as exact values whenever suitable.*
☒	☐	For Bayesian analysis, information on the choice of priors and Markov chain Monte Carlo settings
☒	☐	For hierarchical and complex designs, identification of the appropriate level for tests and full reporting of outcomes
☒	☐	Estimates of effect sizes (e.g. Cohen's *d*, Pearson's *r*), indicating how they were calculated

Our web collection on statistics for biologists contains articles on many of the points above.

Software and code

Policy information about availability of computer code

Data collection	No custom software was used.
Data analysis	Mass spectrometry data was analysed using SCiLS lab (Version 2022b). Further statistical modeling was performed in R (version 4.3.0) and GraphPad Prism (version 10.0.3). IMC, IHC and H&E images were analyzed using HALO (Indica Labs, Albuquerque, NM, USA). LC-MS data was acquired on Analyst® TF 1.7.1 (with Analyst® TF 1.7.1 Components for LC Devices) and processed using Sciex MultiQuant (3.0.2) software. In-vitro data was analysed using Sartorius Incucyte software v2022A. Rna sequencing data was processed using FastQC (v0.11.8), Trimmomatic (v0.39), Picard tools (v2.25.1), Salmon (v1.6.0) and R (version 4.2.2) using the DESeq2 package (version 1.38.35). Gene Set Enrichment Analysis (GSEA) was carried out using the clusterProfiler version 4.6.0.<br>The analysis of VISIUM objects in R (base R version 4.3.3) was conducted with the Seurat framework (version 5.1.0). Plotting, memory conservation, statistical evaluation, cell type deconvolutions involved a suite of packages, including gridExtra (2.3), cowplot (1.1.3), purrr (1.0.2), tidyr (1.3.1), tibble (3.2.1), infercnv (1.20.0), ComplexHeatmap (2.18.0), doParallel (1.0.17), iterators (1.0.14), foreach (1.5.2), spacexr (2.2.1), ggsignif (0.6.4), scales (1.3.0), scCustomize (3.0.1), ggplot2 (3.5.1), dplyr (1.1.4), ggpubr, ape (5.8), BPCells (0.1.0), SeuratDisk (0.0.0.9021), hdf5r (1.3.11), curl (5.2.1), reticulate (1.38.0), SeuratObject (5.0.2), sp (2.1-4), patchwork (1.3.0), and SpatialFeaturePlotBlend (loaded from GitHub at https://github.com/george-hall-ucl/SpatialFeaturePlotBlend, commit 32b98cd).

For manuscripts utilizing custom algorithms or software that are central to the research but not yet described in published literature, software must be made available to editors and reviewers. We strongly encourage code deposition in a community repository (e.g. GitHub). See the Nature Portfolio guidelines for submitting code & software for further information.

Data

Policy information about availability of data

All manuscripts must include a data availability statement. This statement should provide the following information, where applicable:

- Accession codes, unique identifiers, or web links for publicly available datasets
- A description of any restrictions on data availability
- For clinical datasets or third party data, please ensure that the statement adheres to our policy

The non-human data generated for this study will be made publicly available in the University of Cambridge data repository (https://www.repository.cam.ac.uk/home).RNA neurosphere sequencing data is available under the following GEO accession number: GSE288836.
R code for generating K means groups in human and sphere MSI data is available in the University of Cambridge data repository (https://www.repository.cam.ac.uk/home) under the file name: 2-20240214-FinalPlots.R.
VISIUM spatial transcriptomics human R code and analysis is available in the University of Cambridge data repository (https://www.repository.cam.ac.uk/home) under the file name seuraT_obj.R.
Human data: The authors declare that the clinical and imaging data supporting the findings of this study are available within the article and its Supplementary Information. The authors defer raw DESI-MSI and clinical MRI data deposition to ensure compliance with legal requirements of the University of Cambridge and Cambridge University Hospitals NHS Foundation Trust and avoid breaching data sharing regulations for human participants. Requests for raw data can be referred to the corresponding author; these will be reviewed within ten working days in consultation with the institutional R&D which will determine the terms of a data transfer agreement between the recipient institution, the University of Cambridge, and Cambridge University Hospitals NHS Foundation Trust.

Research involving human participants, their data, or biological material

Policy information about studies with human participants or human data. See also policy information about sex, gender (identity/presentation), and sexual orientation and race, ethnicity and racism.

Reporting on sex and gender	Gender was not considered in the study design
Reporting on race, ethnicity, or other socially relevant groupings	No data on race or ethnicity was collected as part of the study design.
Population characteristics	No data on population characteristics was collected in this study.
Recruitment	Patient were recruited to the study following review by consultant Neurosurgeon in the outpatient setting. Only patients suitable, in terms of their general health and co-morbidities, to undergo tumour resection and general anaesthetic were recruited to the study. In addition, MRI was used to select patients with tumours amenable to surgical resection and intra-operative sampling (non-eloquent brain areas, minimal necrosis/cystic component).
Ethics oversight	The study was approved by East of England - Cambridge East Research Ethics Committee - REC 18/EE/0283. Protocol was approved by Cambridge University Hospitals, Addenbrooke's Hospital, Cambridge.

Note that full information on the approval of the study protocol must also be provided in the manuscript.

Field-specific reporting

Please select the one below that is the best fit for your research. If you are not sure, read the appropriate sections before making your selection.

☒ Life sciences ☐ Behavioural & social sciences ☐ Ecological, evolutionary & environmental sciences

For a reference copy of the document with all sections, see nature.com/documents/nr-reporting-summary-flat.pdf

Life sciences study design

All studies must disclose on these points even when the disclosure is negative.

Sample size	Multiple samples were taken from 3 patients with brain tumours that had been infused with [U-13C]glucose. GB1, 40 samples from 10 different regions. GB2, 30 samples from 6 different regions. Metastasis patient, 30 samples from 7 different regions. The determination of sample number per patient were not-predetermined. Rather, the number was guided by intra-operative conditions and patient safety. E.g. localised bleeding following sample collection, time restraints to minimise general anaesthetic, proximity to eloquent brain areas. Thirty- cell lines, derived from 26 different patients, were analyzed as neurospheres. The number of cell lines derived was random.
Data exclusions	Nil
Replication	Several human GB tumour sections and slides were generated for the experiment. Because these were consecutive sections from a tumour, they could be classed as biological replicates. Two separate MSI runs were then performed on the consecutive sections to confirm the metabolic group presence across multiple batches. For the neurosphere experiment, each matrigel dome contained a single cell line with

	multiple (>5) neurospheres from different passages and frozen vials. These therefore served as both technical and biological replicates. All in vitro work included 6 technical replicates and 3 biological replicates.
Randomization	Samples were positioned randomly on slide for Mass Spectrometry Imaging. During analysis all samples were assigned random numbers so as not to bias analysis. There was no group allocation performed prior to analysis.
Blinding	Data analysis was blinded to region of acquisition and patient sample. All GB sections were assigned a random number and processed and analysed in one batch.

Reporting for specific materials, systems and methods

We require information from authors about some types of materials, experimental systems and methods used in many studies. Here, indicate whether each material, system or method listed is relevant to your study. If you are not sure if a list item applies to your research, read the appropriate section before selecting a response.

Materials & experimental systems

n/a	Involved in the study
☐	☒ Antibodies
☐	☒ Eukaryotic cell lines
☒	☐ Palaeontology and archaeology
☐	☒ Animals and other organisms
☐	☒ Clinical data
☒	☐ Dual use research of concern
☒	☐ Plants

Methods

n/a	Involved in the study
☒	☐ ChIP-seq
☒	☐ Flow cytometry
☒	☐ MRI-based neuroimaging

Antibodies

Antibodies used

IMC antibodies:
αSMA 141Pr 1A4 1:50 3141017D Standard Biotools
Vimentin 143Nd RV202 1:100 3143029D Standard Biotools
CD31 145Nd JC/70A 1:50 ab264090 Abcam
Cleaved Caspase 3 147Sm E83-77 1:50 ab208003 Abcam
Pan-CK 148Nd C11 1:50 3148020D Standard Biotools
CD45 152Sm D9M8I 1:100 3152018D Standard Biotools
CD3 154Sm UCHT1 1:50 3170022D Standard Biotools
CD4 156Gd RPA-T4 1:50 3156036D Standard Biotools
CD68 159Tb KP1 1:100 3159035D Standard Biotools
CD8 162Dy RPA-T8 1:50 3162036D Standard Biotools
Ki67 168 Er B56 1:50 3168022D Standard Biotools
Collagen I 169Tm Polyclonal 1:100 3169023D Standard Biotools
Granzyme B 167 ER GB11 1:50 3167023D Standard Biotools
p53 165 DO-7 1:50 MA5-12557 Thermo Fisher
IHC antibodies:
Ki67 Dako M7240 1:1000
CC3 Cell Signaling Technology, 9664 1:250
CD31 Abcam, ab182981, 1.128ug/ml

Validation

Validation was performed as per manufacturer's instructions available online. All antibodies were validated using positive (in tissue known to express high levels of protein of interest) and negative controls in normal and tumour tissue. The IHC antibodies were tested using a pre-determined Bond protocol, using both sodium citrate and Tris EDTA HIERs, no primary controls for each retrieval and a range of antibody dilutions. Custom IMC antibodies were tagged with lanthanides in house, using Fluidigm labeling kits, according to the manufacturer's instructions. All IMC antibodies were validated in house using conventional IHC as well as corresponding IMC staining and assessed by a pathologist.

Eukaryotic cell lines

Policy information about cell lines and Sex and Gender in Research

Cell line source(s)	Human-derived cell lines were sourced from the ICARUS biorepository (REC 18/EE/0172).The following primary human GB cell lines were used in the study: GTP2med, GTP2lat, AT8C1, AT8C2, AW43, AW40, AW42, J3T1, SP20, S2, S1, 64, A11, 57, 64, 69, J5T1, 58, 66, AT20, AT21ant, AT21post, AT5, J20, SP13, AT2, AT3, At12, 48, J5T2
Authentication	STR authentication.
Mycoplasma contamination	All cell lines tested negative for mycoplasma.

Commonly misidentified lines (See ICLAC register)	None. All cell lines were primary human cell lines and were STR authenticated.

Animals and other research organisms

Policy information about studies involving animals; ARRIVE guidelines recommended for reporting animal research, and Sex and Gender in Research

Laboratory animals	Female athymic RNU rats (Charles River). All animals were 6-8 weeks old at the time of tumour orthotopic implantation.
Wild animals	No wild animals were used in the study.
Reporting on sex	All athymic rats used in the study were female. Sex was not considered as part of the study design.
Field-collected samples	No field- collected samples were used in the study.
Ethics oversight	Procedures were performed in compliance with personal and project licenses issued under the United Kingdom Animals Scientific Procedures Act (1986) and approved by the Cancer Research UK, Cambridge Institute Animal Welfare, and Ethical Review Body.

Note that full information on the approval of the study protocol must also be provided in the manuscript.

Clinical data

Policy information about clinical studies

All manuscripts should comply with the ICMJE guidelines for publication of clinical research and a completed CONSORT checklist must be included with all submissions.

Clinical trial registration	REC 18/EE/0172 for cell lines, REC 18/EE/0283 for infusion studies
Study protocol	Three patients from Addenbrooke's Hospital, Cambridge, were infused with [U-13C] glucose. The selection criteria included first clinical presentation and MR images consistent with GB as well as no significant co-morbidities, particularly diabetes mellitus. On the day of the surgery, following induction of anaesthesia, a pyrogen free 5% solution of [U-13C]glucose in sterile saline (Merck, Frankfurter Strasse, Germany) was administered as bolus of 8 g over 10 minutes followed by 8 g/h continuous infusion, as described previously for GB patients and several other tumour types. Arterial blood was collected via a peripheral arterial line prior to bolus administration and then every 15 min following the start of infusion and at 15 min after the end of the infusion. Tumour sampling was guided by intra-operative Stealth navigation, and assessment of 5-ALA fluorescence. Tumours were sampled between 90-150 min as this had been shown previously to be the approximate time at which there was steady state 13C enrichment of plasma glucose. A pituitary ronguer was used to transfer tumour samples directly into liquid nitrogen (LN2). The freezing time was <5 seconds between tissue devascularisation and immersion in LN2.
Data collection	Data was collected via patients recruited at Addenbrooke's Hospital, Cambridge.
Outcomes	N/A

Plants

Seed stocks	NA
Novel plant genotypes	NA
Authentication	NA
